# Feasibility and acceptability of two remote physical activity programs for older Latino adults: the C.E.R.E.B.R.O. study, a pilot randomized trial

**DOI:** 10.3389/fmed.2026.1862017

**Published:** 2026-08-28

**Authors:** Jocelyn Ocampo-Mota, Yuliana Soto, Miguel A. Negrete, Eduardo Cardiel, Brynne Karlinsey Skidmore, Mariana Pinto, David X. Marquez

**Affiliations:** 1 Department of Kinesiology and Nutrition, University of Illinois Chicago, Chicago, IL, United States; 2 Department of Medicine, University of Illinois Chicago, Chicago, IL, United States

**Keywords:** feasibility, Latinos (U.S.), older adults, physical activity, remote

## Abstract

**Introduction:**

Expanding access to culturally relevant physical activity (PA) programs is key to supporting healthy brain aging among older Latinos. The C.E.R.E.B.R.O. study compared two Spanish-language PA programs delivered remotely to Latinos with subjective cognitive impairment. BAILAMOS™ was a 6-month Latin dance-based intervention, and ¡En Forma y Fuerte! (Fit & Strong!) was a 3-month multicomponent program.

**Methods:**

This pilot randomized trial took place remotely via Zoom. Participants were healthy, inactive, and Spanish speaking (aged 60-82). Participants were randomized via computer-generated block randomization to either BAILAMOS™ or ¡En Forma y Fuerte!. The objective of the trial was to compare feasibility of both remote interventions. Feasibility was assessed via (1) reach, (2) recruitment rate, (3) adherence to intervention, (4) retention, and (5) adverse events. Acceptability was assessed quantitatively and qualitatively via a post-program evaluation survey of program components.

**Results:**

The C.E.R.E.B.R.O. study reached 401 individuals, screened 199 individuals, and enrolled & randomized 38 participants (mean age = 68.89 ± 9.74 years; 75% female). The recruitment rate was 9.48%. Nineteen participants were randomized to BAILAMOS™ and nineteen to ¡En Forma y Fuerte!. Mean attendance for BAILAMOS™ was 55.51% across 48 sessions, while mean attendance for ¡En Forma y Fuerte! was 68.42% across 24 sessions, falling under our pre-determined benchmarks. Retention rates were 84.21% for BAILAMOS™ and 89.47% for ¡En Forma y Fuerte!, meeting our success criteria. Program evaluations highlighted high satisfaction, with higher instructor ratings within ¡En Forma y Fuerte!. No serious adverse events were reported.

**Discussion:**

Both remotely delivered, culturally tailored PA programs demonstrated strong retention, high participant satisfaction, and acceptable safety among older Latino adults with subjective cognitive impairment. Although recruitment and adherence remained challenging, higher attendance in the shorter-duration program suggests intervention length may influence sustained engagement. These findings support the potential of remote PA programs to increase access among older Latino adults and highlight the need for improved recruitment and engagement strategies in future trials.

**Clinical Trial Registration:**

https://clinicaltrials.gov/study/NCT05588778?term=NCT05588778&rank=1, identifier [NCT05588778].

## 1 Introduction

Older Latinos are disproportionately impacted by Alzheimer’s disease and related dementias (ADRD), with a risk approximately 1.5 times higher than non-Latino White adults (1). One concerning early symptom reported is subjective cognitive decline (SCD), which is associated with increased risk for future cognitive decline and ADRD (2). SCD is defined as the self-reported decline in cognitive status in comparison to a previous normal state (3). Evidence from studies amongst middle-aged and older community-dwelling Latino adults supports the association between subjective cognitive decline and cognitive decline over time (4). Among Latinos, however, memory concerns may be underreported or normalized due to cultural beliefs, language barriers, and limited access to cognitive health services (5). This is important to consider as in 2021, Latinos represented nearly 19% of the United States population, and this group is projected to grow substantially, with the number of older Latinos expected to reach 19.9 million by 2060 (6). This population growth adds to the urgent need of developing and implementing accessible ADRD risk-reduction strategies tailored for this population.

Physical activity (PA) is an effective non-pharmacological strategy for reducing chronic disease risk (7) and is also recognized as an evidence-based approach for reducing risk of cognitive decline and ADRD (8). Observational data has shown that both light- and moderate-to-vigorous PA associated with higher cognitive performance in domains including verbal fluency and executive function among older Latinos (9). Despite these benefits, many older Latinos engage in low levels of PA (10). Contributing factors may include caregiver responsibilities, limited transportation, and reduced access to recreational facilities (11, 12). Despite this elevated risk, few culturally tailored PA interventions have specifically targeted older Latinos with subjective memory complaints, particularly during the preclinical stage when prevention strategies may have the greatest impact. Given the unique social, cultural, and structural barriers that may limit PA participation, culturally tailored programs may be particularly important for increasing engagement in risk-reduction behaviors and expand access to prevention strategies among older Latinos with memory concerns (13).

There has been growing evidence of culturally tailored PA interventions delivered in-person support cognitive health among older Latino adults (13). The BAILAMOS™ evidence-based dance program highlights the potential of structured, culturally meaningful movement to support PA adherence and cognitive health among older Latinos (13). In a large randomized controlled trial of the BAILAMOS™ dance program led to significant improvements in working memory among middle-aged and older Latinos, with increases in PA mediating these cognitive benefits (14). Earlier pilot work of the in-person version of BAILAMOS™ improved episodic memory compared to a health education control group, suggesting that structured, culturally relevant movement may positively influence cognitive domains that are sensitive to early decline (15). Additional findings from a small study in older Latino adults (>60 years) found that participants randomized into BAILAMOS™ significantly increased their moderate-vigorous PA and showed large improvements in global cognition compared to those in a health education group (13).

Moreover, the BAILAMOS™ dance program has shown to be highly acceptable among Older Latinos with and without cognitive impartment. It was developed in collaboration with a Latino dance instructor and delivered in-person among low-active Spanish-speaking older Latino adults. Formative focus groups indicated that dance was perceived as an accessible and enjoyable form of PA, that is a culturally relevant form of movement introduced at a young age for many Latinos, especially for women who may not be used to participating in traditional exercise (16). One BAILAMOS™ trial with older Latino adults with mild cognitive impairment (MCI) in an adult wellness center (AWC) indicated high acceptability of the program, citing enthusiasm and enjoyment for dance (17). However, participants cited limitations to adherence, including caregiving responsibilities and competing events at the AWC. Evidence suggests that BAILAMOS™ has demonstrated to be acceptable, increase PA, and enhance cognitive functioning, with need for adaptations to increase PA, such as remote delivery. Another evidence-based exercise and health education program that was culturally adapted is the Spanish-language version of Fit & Strong!, ¡En Forma y Fuerte! (18). It was originally developed in English for older adults with osteoarthritis (OA) and is recommended by the Centers for Disease Control (CDC) for individuals with OA (18). ¡En Forma y Fuerte! was culturally adapted for Spanish-speaking older adults with OA. The program was translated and modified to include dancing and Latin music (19).

Evidence suggests that Fit & Strong! has significant improvements in OA and pain management. In a randomized control pilot of 150 participants, Fit & Strong! demonstrated significant improvements in self-efficacy for PA, lower extremity stiffness, and PA engagement that was maintained at 12 months (19). Additionally, En Forma y Fuerte! has yielded significant improvements in lower extremity strength, aerobic endurance, and self-efficacy for exercise. Despite this, Fit & Strong! and ¡ En Forma y Fuerte! have not been assessed for possible cognitive impacts.

Both the English and Spanish in-person interventions have demonstrated feasibility and effectiveness among older Latino adults (18). An 8-week feasibility study among older Spanish-speaking adults with arthritis (*n* = 37) had strong program attendance, and acceptability (18). Participants cited satisfaction with the health education component and variability of the exercise components (18). Although the feasibility of the in-person English & Spanish-version of Fit & Strong! is well supported, there is limited information on the feasibility of the Spanish-version in older adults with subjective memory complaints delivered online.

In-person implementation of both the BAILAMOS™ and ¡En Forma y Fuerte! programs has not occurred without limitations (12, 18). Transportation has been consistently identified as a major barrier, with many older adults relying on others to attend sessions, which can limit participation and adherence. These challenges were further intensified during the COVID-19 pandemic, when older adults faced heightened health risks and reduced access to in-person programming (20, 21). At the same time, the pandemic created an opportunity to adapt established, effective in-person interventions for remote delivery.

Delivering PA programs online may reduce transportation-related barriers, expand reach, and be more accessible among older adults who may benefit from risk-reduction strategies. Participation in one web-based PA intervention suggested that it was an acceptable alternative to in-person PA by community-dwelling older adults (22). Remote PA programs that incorporated direct remote contact, providing live feedback on exercise, demonstrated more favorable outcomes to remote programs that lacked direct contact and comparable PA outcomes to center-based exercises (23). Although these findings are promising, literature demonstrating the feasibility of Spanish-language PA programs for older Latino adults with subjective memory concerns is limited.

Although BAILAMOS™ and ¡En Forma y Fuerte! differ in content, both evidence-based interventions were developed to promote physical activity among older Latino adults and have demonstrated feasibility when delivered in person. BAILAMOS™ uses culturally relevant dance as the primary mode of PA, whereas ¡En Forma y Fuerte! combines traditional aerobic and resistance exercise training with health education and behavior change strategies. Examining the remote delivery of these two culturally tailored, yet distinct, intervention approaches may provide insight into the feasibility and acceptability of different strategies for engaging older Latino adults with subjective memory complaints in physical activity programming.

Feasibility of delivering BAILAMOS™ and ¡En Forma y Fuerte! *remotely* to older Latino adults, particularly those with subjective memory complaints, remains unclear. The primary aim of the Cognitive Enhancement and Risk-Reduction through Exercise for Brain-Related Outcomes Study (C.E.R.E.B.R.O.) was to evaluate the feasibility and acceptability of two remote culturally relevant PA interventions, BAILAMOS™ ¡and En Forma y Fuerte!, when delivered remotely to older Latinos with subjective memory complaints using pre-determined benchmarks, including adherence (≥75%). By examining both a culturally relevant dance intervention and a more traditional exercise-based intervention, this study sought to generate preliminary insights regarding the remote delivery of different PA approaches within this population. We anticipated that remote delivery would be feasible and acceptable, meeting our established benchmarks.

## 2 Methods

### 2.1 Study design

The C.E.R.E.B.R.O. study was a pilot randomized controlled trial designed to assess and compare the feasibility and acceptability of two remote delivered PA interventions, BAILAMOS™ and ¡En Forma y Fuerte!, among older Latino adults with memory concerns. The rational for the selection and comparison of BAILAMOS™ and ¡En Forma y Fuerte! were because both are evidence-based culturally tailored PA programs that have previously demonstrated feasibility and acceptability when delivered in person to older Latino adults. However, the feasibility and acceptability of delivering these interventions remotely, particularly among older Latino adults with subjective memory complaints, had not been established. The feasibility of Fit & Strong! or En Forma y Fuerte! in older Latinos with SMC has never been explored, creating additional rationale to compare the two programs. Additionally, the interventions represent two distinct approaches to promoting physical activity: BAILAMOS™ uses culturally relevant Latin dance, whereas ¡En Forma y Fuerte! combines traditional exercise training with health education and behavior change strategies. Participants were randomized at the end of baseline data collection to one of the two interventions to evaluate the feasibility and acceptability of remote delivery across both programs.

Participants were enrolled and interventions were delivered across two consecutive study waves. The two-wave approach allowed recruitment, intervention delivery, and follow-up assessments to occur in manageable cohorts while maintaining the same study procedures across both waves. Feasibility was assessed using pre-established benchmarks, including recruitment (100 participants enrolled), retention (≥75% retained), adherence (≥75% with ≥80% of intervention sessions completed across both waves), safety (zero unexpected adverse events (AEs) that are serious or study related). Acceptability was assessed qualitatively & quantitatively via a post-program evaluation survey of program components.

### 2.2 Sample

Inclusion criteria included (1) age ≥60 years old; (2) self-identification as Latino/Hispanic; (3) Spanish-speaking; (4) participation in <2 days/week of aerobic exercise; (5) mild cognitive impairment, defined as a score of 18–25 on the Montreal Cognitive Assessment (MoCA) or the presence of subjective memory complaints (responding “Very often,” “Often,” or “Sometimes” to the questions: “About how often do you have trouble remembering things?” from the Rush Alzheimer’s Disease Center); (6) dancing <2 times per month over the past 12 months; (7) willingness to be randomly assigned to either study group; (8) no plans to leave the country for more than two consecutive weeks during the 6-month study period (24, 25). Participants that did not own a tablet were provided with one, but the research team was unable to provide internet access, thus making them ineligible. Eligibility criteria were modified twice in Dec-2023 to accelerate recruitment and be more inclusive of the target population: (1) lowering age criteria from ≥65 to ≥60 and (2) participation in frequency of aerobic exercise from <1 day/week to <2 days/week. Lowering the age criteria made it so that older Latino adults were still targeted but expanded the eligibility pool. Older Latinos are also more likely to experience a higher allostatic load and may experience cognitive complaints earlier in life (26). Changing the criteria for days of aerobic exercise per week made it so that the study still excluded older Latinos that were already active but increased the number of eligible participants.

Exclusion criteria included needing a caregiver for daily functioning, self-reported presence of uncontrolled cardiovascular disease or uncontrolled diabetes mellitus, pacemaker *in situ*, stroke, severe chronic obstructive pulmonary disease (COPD), and recent healing or unhealed fracture(s).

The Exercise Assessment and Screening for You (EASY) tool was used during the initial eligibility screening call. Participants that reported feeling unsteady or always using an assistive device were excluded from the study. If a participant reported feeling unsteadiness while standing or walking, they were required to get medical clearance before enrolling. If a participant required medical clearance, contact information for their primary medical care provider was collected by the study team member completing the screening call. The study coordinator contacted participant’s primary medical providers to obtain a signed copy of the medical release form via fax.

### 2.3 Recruitment and study procedures

Recruitment and delivery of study interventions were done in two consecutive waves. Each wave consisted of (1) recruitment period, (2) enrollment & randomization, (3) program delivery, and (4) testing. Recruitment for the first wave began in May, 2023 and concluded mid-October, 2023. Participants from the first wave were randomized to BAILAMOS™ or ¡En Forma y Fuerte! via computer-generated block randomization in block sizes of eight. Both programs ran parallel to one another and began on October, 2023. Any eligible participant that enrolled after were randomized to the wave 2 cohort. Research assistants were blinded to participant’s randomization groups.

Recruitment for the second wave began on October, 2023 and concluded on March, 2024. Despite having enrolled less than 100 participants, recruitment for both waves was suspended as it exceeded the planned allotted time, to allow sufficient time to deliver programming and complete data collection. Wave 2 programs also ran parallel to one another and began in March, 2024. Both interventions were delivered concurrently during each wave, allowing feasibility and acceptability outcomes to be evaluated across two culturally tailored physical activity programs delivered remotely. The pilot trial ended on October, 2024 after completion of 2 waves of recruitment and intervention delivery.

Most study procedures were performed remotely, except for recruitment. Older Latino adults were primarily recruited through health/wellness fairs, senior centers, churches, and community events. The study team included a community liaison who identified possible recruitment sites and conducted participant recruitment alongside other bilingual research team members. Many of these team members, including the community liaison, were also bicultural/Latino. During in-person events, study team members distributed study flyers and collected interest/permission-to-contact forms. When allowed, the study team made announcements during Spanish-language masses and included study information in a church’s weekly bulletin. In addition to attending in-person events, the study team distributed flyers at senior centers and local businesses. Enrolled participants could also refer someone they knew to the study. If the referral yielded a successful enrollment (defined as signing the informed consent), the participant that made the referral received compensation via a $10 gift card.

After an in-person recruitment event five bilingual and bicultural research assistants, who were CITI certified and had received training from the study PI in plain-language communication, contacted individuals that signed an interest form via telephone. During these calls, key components of the study (e.g., online programming) and participant questions were thoroughly discussed. If the participant was still interested, the research assistant administered the eligibility screening. If eligible, participants were scheduled for a separate remote informed consent and baseline data collection call via telephone. Additionally, during the eligibility screening, research assistants identified participants that would need a study tablet.

During the baseline data collection, research assistants first reviewed the informed consent document and then discussed questions or concerns with the participant. After, participants signed the informed consent electronically via REDCap and then proceeded with the baseline data collection (27, 28). Participants were randomized only after informed consent and baseline data collection were completed. Participants were delivered all intervention and evaluation materials (e.g., En Forma y ¡Fuerte! program manuals, ankle weights, and post-program evaluation surveys, tablet and zoom guides) via the United States Postal Service (USPS). Participants who did not have access to a compatible device were also provided with a study tablet. Tablets were set up prior to being mailed to participants, including setting the language to Spanish and organized the home screen to easily access Zoom.

Prior to the start of the intervention, participants completed at least one one-on-one remote orientation session to learn how to use their device and access the Zoom platform. These sessions were completed with a research assistant who was Latino and bilingual via telephone. Basic tablet gestures (e.g., swiping up/down) were covered. Tablets were set up prior to being mailed to participants. The research team set tablet language to Spanish and organized the home screen so that only important apps, like Zoom, appeared. Research assistants guided participants on how to access the Zoom room and practice basic controls like muting and unmuting. Average phone calls were 1 h in length. Participants additionally received a printed step-by-step guide for how to access the Zoom application from their study device, see Supplementary File 1.

Post-program assessments were conducted remotely via telephone at 3 and 6 months after the start of the intervention. Participants received a $15 gift card for each testing session complete. Program evaluation surveys were mailed to participants via USPS, who were asked to complete and return them by mail. Additional details regarding study procedures are described elsewhere (29). The UIC Institutional Review Board approved this study: STUDY 2022-1086.

### 2.4 Physical activity interventions

BAILAMOS™ and ¡En Forma y Fuerte! were delivered in Spanish by bilingual instructors. Both interventions met via Zoom twice a week. The C.E.R.E.B.R.O project coordinator created and managed the program Zoom rooms and distributed login info to participants and instructors. During intervention delivery, Zoom rooms were opened approximately 30 min before every class to allow participants adequate time to log on. If participants needed assistance with logging on, they could call a research team member via telephone or sometimes had family members at home that could also assist. Participants kept their cameras on at all times and two research assistants were present during each class session. One team member offered technical assistance while the other monitored participants while they exercised. Attendance was recorded by a research assistant during each session. Any important notes or events related to intervention delivery and participant engagement were documented by research assistants.

Class sessions for both programs were recorded and uploaded to a secure Box health data folder. Participants that could not attend a live class session were sent that days recording to watch at a later time. If a participant watched the recording, they were instructed to complete the dance or exercise log for that session. If a dance or exercise log was collected for that session, they received the attendance point for that class session.

#### 2.4.1 The adapted remote version of the BAILAMOS™ dance program

The remote BAILAMOS™ program was adapted from the original in-person intervention to allow participation from home while maintaining the core dance and health education components. The program was delivered over 6 months and consisted of twice-weekly 60-min sessions that included a warm-up (5–10 min), a dance segment (40 min), and a cool-down (5–10 min), for a total of 48 sessions and approximately 54 h of program content.

To facilitate remote delivery, partner dancing, a component of the original in-person intervention, was removed and participants completed all dance activities independently within their homes. No additional modifications were made to the program curriculum or progression of dance styles.

Both BAILAMOS™ waves were led by professional dance instructors MM and MT. MM, co-creator of BAILAMOS™, taught the first 4 months of the intervention and had previous experience delivering the program. MT taught the final 2 months and received training on program administration, including class structure and implementation of warm-up and cool-down activities.

Warm-up and cool-down stretches included: shoulder shrugs, sunbursts, heel lifts, toe raises, and ankle circles. A new dance style was covered each month and included Merengue, Cha Cha Cha, Bachata, Salsa, Cumbia, and Kizomba. Each month began with covered basic steps (e.g., Basic step forward) first, followed by more advanced moves (e.g., full and half turns) as sessions progressed. Each dance segment followed a consistent format. In which participants first reviewed dance moves without music, practiced with music, and took a brief break if needed. Participants were encouraged to ask questions throughout the session, and instructors demonstrated movements at a slower pace. Music was played by a research team member using the share audio feature on Zoom.

At the end of each session, participants completed a dance log which recorded number of minutes danced and assessed how they felt before, during, and after the class. In addition, participants attended a monthly 60-min health education discussion held during the first session of each month for a total of six sessions. Guided by Social Cognitive Theory, these discussions focused on promoting PA and were led by a member of the research team. Additional details of the BAILAMOS™ program are described elsewhere (16).

For BAILAMOS™, fidelity checks were completed during the second session of each new dance style. Research assistants observed classes live and completed a fidelity checklist that assessed warm-ups, dance moves, and cool-downs. Deviations from the class structure or program calendar were documented. After fidelity checks, the program instructor received feedback.

#### 2.4.2 The adapted remote version of the ¡En forma y Fuerte! program

The ¡En Forma y Fuerte! program was delivered over 3 months and consisted of twice-weekly 90-min sessions, for a total of 24 sessions and 36 h of program time. Each session included 60 min of exercise, consisting of a warm-up (10 min), an aerobic portion (5–10 min), chair strength exercises focused on lower-extremity strength (35–40 min), and a cool-down (10 min). The intervention was adapted for remote delivery via Zoom while preserving the core exercise and health education components of the original program. To enhance safety during remote participation, floor-based exercises included in the original in-person program were removed. No other substantive modifications were made to the intervention content or behavior change curriculum.

The ¡En Forma y Fuerte! waves were led by bilingual exercise instructors LJ and AT. LJ, who taught the first wave, was a certified group fitness instructor through the American Council on Exercise (ACE), while AT, who taught the second wave, was a certified personal trainer through the National Academy of Sports Medicine (NASM). Both instructors completed the official ¡En Forma y Fuerte! instructor orientation training provided by the Fit & Strong! management team prior to program delivery.

Warm-ups and cool-downs involved neck, trunk, shoulders, and extremity stretches. The aerobic portion of class consisted of a combination of low-impact aerobics movements, stepping in place, and dancing. Music was played during this portion by a research assistant using the share audio feature on Zoom. The lower body strength exercises consisted of six core exercises: seated knee extensions, standing side leg lifts, standing back leg lifts, standing knee lifts, standing hamstring curls, and squats. Ankle weights were used during every class session and were adjustable in ½ lb increments. Each participant had an individualized starting weight, half of the max weight they could lift at one time, that was determined on the first day of class. As the program progressed, participants who completed all repetitions and sets in good form were encouraged to increase their ankle weight.

During class sessions, the exercise instructor first demonstrated how to perform the exercise move. Then, while participants exercised, the instructor monitored them to ensure they were completing the move in good form and provided feedback. The remaining 30 min were dedicated to a behavior change discussion. These sessions were also guided by Social Cognitive Theory and emphasized constructs such as self-efficacy. The health education topics were outlined in a participant manual that was mailed to study participants. Participants were able to follow along with discussion topics using the manual. At the end of each class session, participants completed an exercise log which recorded time spent on each exercise component, number of repetitions, sets, weight, and exertion level.

The 3-month version of ¡En Forma y Fuerte! was selected because feasibility and efficacy of the in-person program had previously been established using this intervention duration. Additional details of the ¡En Forma y Fuerte! program are described elsewhere (20).

For ¡En Forma y Fuerte!, a recording of the first-class session was submitted to the Fit & Strong! Management team who completed a fidelity checklist that assessed warm-ups, exercise components, cool-downs, and any deviations from class structure. After completion of the fidelity check, feedback was provided to the program instructor.

### 2.5 Feasibility and acceptability measures

Feasibility was assessed using reach, recruitment, adherence, retention and safety, and acceptability.

#### 2.5.1 Reach and enrollment

Reach was defined as the total number of individuals reached during recruitment and was documented and defined as the number of individuals that had either: (1) signed an interest form at a recruitment event, (2) contacted the research team directly, or (3) were referred to the study by an enrolled participant. Enrollment was defined as having signed the informed consent document via REDCap (27, 28), which took place during the baseline data collection session. The target benchmark was to enroll 100 participants. The C.E.R.E.B.R.O. study was not meant to be a fully powered randomized control trial, but the target benchmark of 100 participants was selected to be a large enough sample for statistical analysis.

#### 2.5.2 Recruitment rate

Recruitment was calculated as the total number of participants enrolled over total reached ×100.

#### 2.5.3 Participant retention

Retention rate was calculated as the total number of participants who completed post-program assessments over the total number of participants who enrolled ×100, per intervention group, with a target goal of ≥75 % retention.

#### 2.5.4 Adherence to intervention

Adherence to intervention was assessed as the number of sessions attended over total possible session ×100, per intervention group, with a target goal of ≥ 80 % of intervention sessions completed.

If a participant missed a live class session but watched the recording and completed the dance/exercise log for that session, they were logged as having attended that session.

#### 2.5.5 Safety

Safety was assessed via adverse events and were defined as any unexpected or harmful medical occurrences experienced by a participant during their involvement in the study. Any adverse events that were serious or study related were documented and reported to the university IRB by the study coordinator. Adverse events were documented through the length of the entire study and reported as soon as study personnel were made aware. The feasibility threshold for adverse events was 0 events that were serious or study related.

#### 2.5.6 Acceptability

Acceptability of both programs was assessed quantitatively and qualitatively via post-program evaluations tailored to each intervention’s program components, which included multiple choice and open-ended questions. Both evaluation questionnaires assessed participant’s attitudes toward the program instructor and the difficulty, progression, and enjoyability of exercise/dance moves. The ¡En Forma y Fuerte! program evaluation also assessed attitudes toward the participant manual, the group discussion components, and the remote aspect of the program; These items were not covered in the BAILAMOS™ post-program evaluation.

The BAILAMOS™ evaluation had 15 items on a 4-point Likert scale (1 = strongly disagree, 2 = disagree, 3 = agree, 4 = strongly agree and 1 = poor, 2 = fair, 3 = good, and 4 = excellent) and four open response items. The ¡En Forma y Fuerte! post-program evaluation had 27 items on a 5-point Likert scale (1 = strongly disagree, 2 = disagree, 3 = neither agree nor disagree, 4 = agree, 5 = strongly agree) and eight open response items. For each post-evaluation questionnaire, the sum of all items was added together and divided by the total possible scores ×100. Additionally, for ease of comparison, similarly worded items across both questionnaires (e.g., the exercises progressed at an appropriate pace for me), mean scores were calculated. Higher scores indicated higher participant satisfaction. Acceptability was not outlined in our published protocol but was included as an additional measure to assess participants attitudes toward programming.

### 2.6 Statistical analyses

For the purpose of this study, descriptive statistics were performed using SPSS version 29.0.1.0 (IBM Corp., NY, USA). Continuous variables are presented as means and standard deviations, while categorical variables are presented as frequencies and percentages.

Qualitative responses from open-ended items on the post-program evaluation questionnaires were analyzed using reflexive thematic analysis (30). Two members of the research team (JOM, YS) reviewed participant responses, generated initial codes, and collaboratively identified patterns and themes across responses. Themes were refined through discussion and consensus among the coders and were used to summarize participant perceptions of the programs.

## 3 Results

A total of 38 participants enrolled and were randomized to one of two intervention conditions. Participants had a mean age of 68.89 ± 9.74 years, 95% were female, and demonstrated mild cognitive impairment (mean MOCA score = 22.38 ± 3.461). Additional participant characteristics are presented in Supplementary File 2.

### 3.1 Reach and recruitment

The C.E.R.E.B.R.O. study recruited participants for 10 months throughout the Chicagoland area, averaging 3.8 participants enrolled per month. A detailed breakdown of recruitment rates month-to-month is found in Table 1. Delays in recruitment in the first 3 months were due to the time required to obtain permission from churches or other community organizations to recruit at their events.

**TABLE 1:** C.E.R.E.B.R.O Recruitment.

Month-Year	Participants enrolled	Participants reached	Recruitment rate (%)	Recruitment events attended
May-2023	0	1	0%	1
June-2023	1	10	10%	1
July-2023	0	1	0%	0
August-2023	3	40	8%	10
September-2023	1	46	2%	8
October-2023	8	63	13%	13
November-2023	7	66	11%	10
December-2023	4	35	11%	8
January-2023	4	74	5%	7
Feburary-2024	5	40	13%	13
March-2024	5	25	20%	3
Total	38	401	9.48%	74

Partial delay for recruitment during initial months was due to gaining permission and access to community events at churches.

**FIGURE 1:**
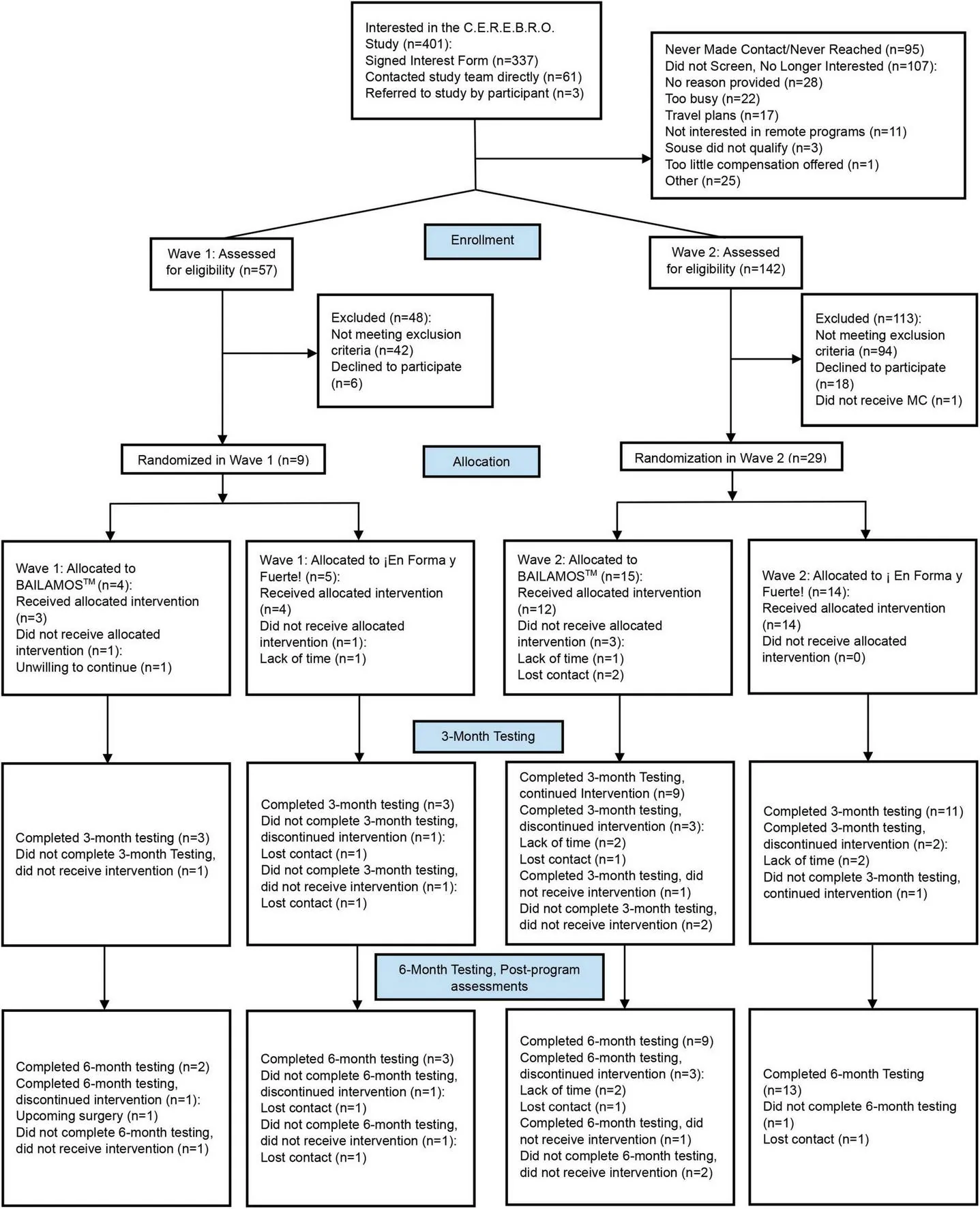
Participant recruitment and retention CONSORT flowchart.

Across 10 months, 401 individuals were reached and expressed interest in the study. The most common method of reaching individuals was through in-person recruitment. Of the 401 individuals reached, the research team was not able to make over the phone contact with 95 individuals, and 107 individuals were reached but were no longer interested in screening (see Figure 1). A total of 199 individuals were screened for eligibility. Of which 136 individuals were deemed ineligible and a total of 38 participants (mean age = 68.89 ± 9.74 years; 75% female) enrolled into the program. The overall recruitment rate was 9.48%.

The most common recorded reason for no longer being interested in screening was being too busy/the study being too much of a time commitment (*n* = 22), travel plans interfering with study timeline (*n* = 17), and not being interested in remote programming (*n* = 11).

### 3.2 Adherence, participation, and retention

Adherence, participation, and retention outcomes for both interventions are presented in Table 2.

**TABLE 2:** Results of BAILAMOS™ and ¡En Forma y Fuerte!.

Item	BAILAMOS™	¡En Forma y Fuerte!
Randomized, n	19	19
Received program, n (%)	15 (78.94%)	18 (94.73%)
Participant Retention, %	84.21%	89.47%
Serious Adverse Events, related to the study	1	0
Acceptability		
Post-program assessments completed (% of total)	10 (52.63%)	14 (73.68%)
M Overall score % ± SD	78.33 ± 10.48	90.69 ± 9.48

*M* mean, *SD* standard deviation

Nineteen participants were randomized to BAILAMOS™ (four in Wave 1, and fifteen in Wave 2). Fifteen received the dance program, while four participants did not (i.e., did not attend sessions). A total of eleven participants completed the BAILAMOS™ intervention, with a mean attendance of 55.51% across 48 sessions, including sessions attended asynchronously. The retention rate for BAILAMOS™ was 84.21% (16 completed post-program assessments/19 participants enrolled).

Nineteen participants were randomized to the ¡En Forma y Fuerte! program (five in Wave 1, and fourteen in Wave 2). Eighteen participants received the exercise program, while one did not attend any sessions. A total of fifteen participants completed the ¡En Forma y Fuerte! program. Mean attendance for ¡En Forma y Fuerte! was 68.42% across 24 sessions, including sessions attended asynchronously. The retention rate for ¡En Forma y Fuerte! Was 89.47% (17 completed post-program assessments/19 participants enrolled).

Reasons for drop out amongst both programs included caregiving responsibilities, programming conflicting with work schedules, and losing contact with participants. Documented session notes showed that some participants received help from family members to access the zoom room. Sometimes participants were unable to log on to class if the family member was not present.

### 3.3 Safety

Three participants experienced reportable AEs, one in BAILAMOS™ and two in ¡En Forma y Fuerte!.

The BAILAMOS™ participant reported back discomfort, related to previously diagnosed osteopenia, that persisted through the length of the program and sometimes caused them to leave class early. The participant discontinued the intervention due to an upcoming surgery related to their osteopenia. The participant was not sure whether the back discomfort was related to a fracture or not. The research team did not have access to the participant’s medical records to confirm.

The two adverse events reported by ¡En Forma y Fuerte! Participants were unrelated to the program.

### 3.4 Acceptability

Twenty-four participants, 10 from BAILAMOS™ and 14 from ¡En Forma y Fuerte!, completed a post-program evaluation survey. See Table 3 for mean scores of program components. For both questionnaires, the sum of all items were added together and divided by the total possible scores ×100. Thus, higher scores were indicative of greater overall acceptance of the programs.

**TABLE 3:** Post-program evaluation item scores.

Post-program evaluation item	BAILAMOS™ mean scores (percentages)	¡En Forma y Fuerte! Mean scores (percentages)
The instructor was enthusiastic and friendly	3.20/4.00 (80.00%)	4.93/5.00 (98.60%)
The instructor helped me adapt the dances/exercises to my level of ability	3.10/4.00 (77.50%)	4.93/5.00 (98.60%)
The instructor clearly explained how to the dances/exercises	3.00/4.00 (75.00%)	2.83/5.00 (56.60%)
The exercises progressed at an appropriate pace for me	3.40/4.00 (85.50%)	4.86/5.00 (97.20%)
The dance routines were fun to do / The class was fun and engaging	3.30/4.00 (82.50%)	4.86/5.00 (97.20%)
I plan to continue doing the BAILAMOS™ dances/*¡*En Forma y Fuerte! exercises on my own	3.00/4.00 (75.00%)	4.77/5.00 (95.40%)
I would recommend BAILAMOS™ to my friends or family members / I would strongly recommend ¡En Forma y Fuerte! @ Home to friends or family	3.30/4.00 (82.50)	96.43/100 (96.43%)

For BAILAMOS™, the total mean score was 47/60 (median = 49.50, SD = 10.48) or 78.33%. Participants had generally positive feedback for both program instructors. Via the open-response questions, one participant cited that it would have been helpful if instructors demonstrated the dance moves from different angles more often. Another participant expressed they did not have enough space to move around in their home.

For ¡En Forma y Fuerte!, the total mean score was 122.43/135 (median = 126.50, SD = 9.48) or 90.69%. Via the open-response questions, participants reported positive feedback on the instructor, stating that the instructor was fun and attentive to their needs. Participants had mixed feelings on technology usage for class. Some participants stated that technical assistance provided by the research staff was helpful, while others expressed difficulty logging on.

Feedback was generally positive across both programs. Participants found dance and exercise moves to be enjoyable, easy to follow, and age appropriate. Participant responses in both programs, however, were mixed in regard to participating at home. Some participants appreciated the flexibility that a remote program offered, while others preferred to be able to leave the house.

Comparing similar items across evaluations, participants across both programs stated that they would recommend the program to friends or family (BAILAMOS™ mean = 3.30, ¡En Forma y Fuerte! mean = 96.43) and that they planned on continuing the dances or exercises on their own (BAILAMOS™ mean = 3.00, ¡En Forma y Fuerte! mean = 4.77). Higher instructor ratings were reported within ¡En Forma y Fuerte! compared to BAILAMOS™ (Table 3). Notably, participant responses highlighted instructors’ demonstration of how to do the dance or exercises moves as an area for improvement, with BAILAMOS™ scoring slightly higher than ¡En Forma y Fuerte! (BAILAMOS™ mean = 3.00, ¡En Forma y Fuerte! mean = 2.83).

## 4 Discussion

The aim of the C.E.R.E.B.R.O. study was to assess the feasibility and acceptability of two Spanish-speaking programs delivered-remotely for older Latinos with subjective memory complaints. Findings suggest that remote delivery of culturally tailored physical activity programs is acceptable and can support participant retention in this population, although challenges related to recruitment and sustained participation remain. Recruitment and adherence in the study did not meet our predetermined benchmarks. However, retention rates and acceptability were high in both BAILAMOS™ and ¡En Forma y Fuerte! groups, with retention rates above the predetermined benchmark of 70% and mean average acceptability scores of participants being 78.33% and 90.69%, respectively. Together, these findings indicate that while participants valued both programs once enrolled, additional strategies are needed to improve recruitment efficiency and ongoing engagement in remotely delivered interventions.

### 4.1 Reach and recruitment

Recruitment was one of the most challenging aspects of the C.E.R.E.B.R.O. study. Although more than 400 individuals were reached through community-based outreach efforts, only 38 participants ultimately enrolled, similar to previous studies reflecting challenges commonly observed in feasibility studies targeting older adults with cognitive concerns (31). These findings highlight the substantial effort required to recruit older Latino adults with subjective memory complaints into remotely delivered lifestyle interventions, particularly when eligibility criteria include cognitive, health, and technology-related requirements. Recruitment progressed slowly during the initial months, partly due to delays in building trust and obtaining permission from churches or other community organizations to recruit at their events. Without prior approval from many community partners, recruitment options were initially limited. This is consistent with the findings of Teufel-Shone et al. (32), which suggested that establishing new partnerships and trust with local community organizations requires substantial time, especially when there is a lack of pre-established connections (32). Additionally, even when community organizations are open to a partnership, delays can result from time constraints of personnel and the organizations prioritizing the timelines of internal projects (33).

Recruitment for the second wave progressed more smoothly, likely reflecting increased community awareness of the study and word-of-mouth, and the benefit of previously established community partnerships. Future studies may benefit from earlier partnerships with healthcare systems or community networks. Leveraging established partnerships or community relationships may accelerate the approval process and allow for earlier access to recruitment sites and events. For example, Ojo et al. worked with a key community leader, an aerobic fitness instructor who was influential and trusted within the target community (34). To recruit for a technology-based fall risk assessment study during the early COVID-19 pandemic. This strategy generated the greatest interest in the study and yielded the most enrolled participants.

Our study similar to previous research, highlight that in-person recruitment strategies, primarily at wellness fairs and church-based events, generated the greatest initial interest (35). However, follow-up after recruitment events proved challenging, as a large proportion of individuals who signed interest forms could not be recontacted. This may be partially attributable to phone calls from unfamiliar numbers, a barrier noted in prior community-based studies (34). Our team provided interested participants with the phone number that a research assistant would call from, but future studies can incorporate in-person screening sessions to further engage participants or encourage interested participants to save the study’s phone number under their contact list. Studies that embedded recruitment within existing registries or provided intensive follow-up support, such as home visits or technology setup assistance, have reported higher enrollment efficiency, though often with greater resource demands (36). Taken together, these findings highlight the importance of building trust and maintaining participant engagement beyond the initial point of contact. Future studies should allocate sufficient time and funding for recruitment and consider strategies that strengthen ongoing communication and trust among older Latino adults.

However, our study’s recruitment rate is comparable to other community-based PA feasibility studies among Latino or Hispanic older adults. For example, Zlatar et al. (37) reported a recruitment rate of approximately 5 participants per month in a fully remote, culturally adapted PA pilot study for middle-aged and older Hispanic adults, with an enrollment rate of 50% among prescreened individuals (37). Although follow up and screening were also conducted via telephone, the higher enrollment rate could be partially attributed to their use of a *promatora de investigación* (community research worker), with established ties to community partners and community health worker network, during recruitment. Although we used a bicultural and bilingual community liaison, who worked to identify possible recruitment sites and events, they did not have any pre-established links to local community partners. The success of this approach highlights the importance of trusted community relationships when engaging Latino populations in research, particularly for studies involving technology use or cognitive health concerns.

Similarly to our study, Bronas et al. (38) screened 69 individuals of which 39 participants enrolled in a remote, technology-supported PA intervention for midlife and older Latino adults, suggesting that recruiting participants for cognitively focused lifestyle interventions may require screening large numbers of individuals to identify eligible participants (38). Taken together, these findings suggest that recruitment challenges observed in the C.E.R.E.B.R.O. study were not unique, but rather reflect broader barriers associated with enrolling older Latino adults into remote, prevention-focused interventions. Older adults have a wide range of technology-based needs and may feel overwhelmed by navigating a new device or platform, creating a barrier to enrollment (39). Future studies may benefit from leveraging trusted community health workers, community leaders, and existing community partnerships to strengthen recruitment efficiency and participant engagement.

### 4.2 Retention and adherence

Despite recruitment barriers, retention was high in both intervention groups, exceeding pre-stablished benchmark of 75%. Retention was 84.21% retention in BAILAMOS™ and 89.47% in ¡En Forma y Fuerte!, suggesting that participants who enrolled generally remained engaged with the study through follow-up assessments. This distinction between retention and adherence is noteworthy, as participants appeared willing to remain involved in the study even when consistent session attendance was difficult. In contrast, adherence fell below the pre-established benchmark for both interventions, with participants *averaging 55.51% of sessions attended in BAILAMOS™ and 68.42% of sessions attended in* ¡*En Forma y Fuerte! including sessions attended asynchronously.* These findings suggest that while the programs were acceptable to participants, barriers remained that limited regular participation in remotely delivered sessions.

Differences in retention and adherence between the 3-month and 6-month programs may be partially attributable to intervention duration rather than acceptability of the remote delivery format. Longer behavioral interventions are known to introduce greater participant burden, which can negatively impact sustained engagement and attendance over time (40). Prior feasibility studies in older Latinos and older adults with MCI and dementia have similarly documented declines in attendance as intervention length increases, even in culturally tailored programs (17, 41). Consistent with these findings, attendance in BAILAMOS™ was the highest in the first 3 months of the program and then declined. Similarly, attendance in ¡En Forma y Fuerte! remained consistent in the first 2 months and saw a small decline in the third and final month of the intervention. Although the present study was not designed to formally compare intervention formats, the observed pattern suggests that intervention duration may be an important consideration when designing remote physical activity programs for older Latino adults with memory concerns. Future studies should explore strategies to sustain engagement throughout longer interventions, such as booster sessions, periodic re-engagement activities, or additional participant support.

Differences in adherence may also reflect variation in opportunities for participant interaction across programs, as well as challenges associated with remote participation. Research suggests that the establishment of social connections and bonding can strengthen overall adherence to PA interventions (42, 43). While remote PA-interventions are convenient and may reduce transportation challenges, they can also limit positive social interactions. During the dance and exercise components of both programs, participants were asked to keep their microphones off to eliminate background noise. Although this preserved audio quality, it reduced the opportunity for participants to engage in the small talk that may foster positive group connection. Yet, the ¡En Forma y Fuerte! discussion component created an opportunity for participants to turn on their mic and interact with one another and the instructor. This additional opportunity for engagement may have contributed to the higher adherence observed in ¡En Forma y Fuerte! and highlights the potential importance of intentionally incorporating social interaction into remotely delivered programs for older adults.

It is also important to consider the potential influence technology-related barriers and memory- concerns on intervention participation (44). For older Latino adults, remote participation may present a two-fold challenge: learning to navigate unfamiliar technology while simultaneously managing memory-related difficulties that may affect logging into sessions, remembering class schedules, or troubleshooting technical issues. A previous study by Cintoli et al. (44) that delivered a remote cognitive program for patients with dementia had an adherence with over 90% and no participant attrition. In their study protocol, prior to delivery of the intervention, participants completed a computer literacy session and had access to ongoing technical support throughout the study, including telephone, email, and chat assistance when difficulties arose. Our team offered orientation sessions prior to the beginning of the intervention, which were held over the phone. Although these sessions were helpful to orient participants to the basic navigation of Zoom, many participants needed support throughout the programs. Some participants also reported that family members assisted them with accessing the program, however support was inconsistent. In-person sessions may be better for strengthening technology confidence and making logging into a remote class more feasible (45). Future studies should embed additional digital literacy protocols, provide booster technology support sessions throughout the intervention, and explore strategies to engage caregivers or family members when appropriate. Taken together, these findings suggest that remote interventions for older Latino adults with memory concerns may require ongoing technology support, simplified access procedures, and opportunities for caregiver or family involvement to facilitate consistent participation.

### 4.3 Acceptability

Acceptability of both remote programs was high, with participants reporting overall satisfaction with the intervention content, instructors, and opportunity to engage in structured physical activity, although some participants reported a preference to exercise outside of their home. Future researchers should consider delivering PA-interventions using a blended hybrid format. Participants valued the group-based format of both programs and expressed interest in greater opportunities for social connection during sessions. A blended format could better promote group cohesion and community, while allowing flexibility when delivered from home. Findings suggest that a hybrid program structure can be well received by older adults, although the availability of a program instructor is still crucial (46).

Qualitative feedback further highlighted the important role of instructors in shaping participant experience. Participants described instructors as encouraging, engaging, and attentive to their needs, particularly in BAILAMOS™, where enthusiasm and encouragement were described as motivating factors.

Responses from both post-program evaluations also suggested that clearly demonstrating how to do a dance or exercise move was an area of possible improvement. Related, findings from other studies reported that demonstrating movements requires added effort compared to in-person interventions. Delivering movement-based interventions remotely may require additional instructor skills compared to in-person programs, including effective camera positioning, verbal cueing, and visual demonstrations. Future researchers should consider instructor training to enhance participant engagement via remote platforms as previously suggested by Dagenais et al. (47). Notably, a hybrid program model may also make it easier for instructors to demonstrate exercise or dance moves in-person and reviewing steps in the online format.

High acceptability despite limited feasibility aligns with prior studies of remote interventions in older adults with cognitive impairment, which have reported positive participant perceptions even when implementation challenges were present (44). The consistently high acceptability observed across both BAILAMOS™ and ¡En Forma y Fuerte! suggests that older Latino adults with subjective memory complaints are receptive to multiple culturally tailored approaches for promoting physical activity in a remote format. However, future interventions should focus on strengthening participant engagement, social connection, and technology support while maintaining the culturally relevant features that contributed to positive participant experiences.

### 4.4 Strengths and limitations

This study is the first to evaluate the feasibility and acceptability of Spanish-language, remotely delivered versions of both BAILAMOS™ and ¡En Forma y Fuerte! among older Latino adults with subjective memory complaints. A major strength is the focus on a culturally tailored, underserved population at elevated risk for cognitive decline. Additional strengths include the evaluation of two culturally tailored physical activity interventions, and the use of evidence-based behavior change programs designed to promote physical activity and support brain health.

Several limitations should be noted. Recruitment did not meet target goals, limiting sample size and generalizability. Adherence was lower than expected, potentially due to intervention length, digital literacy barriers, and limited opportunities for social interaction in a fully remote format. Because the primary aim was to evaluate feasibility and acceptability, the study was not powered to formally compare outcomes between intervention groups, and any observed differences between BAILAMOS™ and ¡En Forma y Fuerte! should be interpreted cautiously. Although all feasibility benchmarks were not met, high participant satisfaction underscores the acceptability of remote physical activity programs within this population.

An additional limitation is that BAILAMOS™ and ¡En Forma y Fuerte! differed in duration and total contact hours (6 months and 54 h vs. 3 months and 36 h, respectively). Therefore, observed differences in adherence and retention may reflect intervention dose rather than differences in program content. Future studies should systematically evaluate the influence of intervention duration and contact hours on feasibility outcomes to identify the optimal balance between participant engagement and intervention burden. Additionally, although participants were enrolled based on subjective memory complaints, the relationship between level of cognitive impairment and study adherence was not examined. Future research should investigate whether cognitive status influences participation, technology use, and retention in remotely delivered physical activity interventions and determine whether additional support strategies are needed for individuals with greater cognitive challenges.

Future studies should consider partnering early with community organizations and healthcare systems to enhance recruitment, embedding technology orientation and booster sessions to support digital access, and exploring hybrid or shorter-duration models to improve engagement. Incorporating structured opportunities for peer interaction may also help strengthen social connection and sustain participation in remote delivered programs.

## 5 Conclusion

The C.E.R.E.B.R.O. study highlight both the promise and challenges of delivering culturally tailored PA programs remotely to older Latino adults with subjective memory complaints. Findings demonstrated that participating in remote PA-interventions is safe and highly acceptable for older Latino adults, with both BAILAMOS™ and ¡En Forma y Fuerte! receiving favorable participant feedback. While feasibility benchmarks for recruitment and adherence were not fully achieved, retention exceeded pre-established benchmarks, suggesting that older Latino adults with memory concerns are receptive to remotely delivered PA interventions once enrolled.

The inclusion of two culturally tailored interventions with differing PA modalities (dance vs. resistance training) provided, differing durations, and contact hours provided preliminary insight into factors that may influence feasibility in this population. Findings suggest that intervention duration, opportunities for social interaction, technology support, and instructor engagement may play important roles in sustaining participation. Future research should focus on optimizing remote delivery strategies, like optimizing instructor training, to enhance feasibility while maintaining the cultural relevance and participant satisfaction observed in this study. Researchers should also consider exploring strategies to strengthen ease and speed of recruitment.

## Data Availability

The datasets presented in this study can be found in online repositories. The names of the repository/repositories and accession number(s) can be found below: clinicaltrials.gov: https://clinicaltrials.gov/study/NCT05588778?term=Cognitive%20Enhancement%20and%20Risk-reduction%20Through%20Exercise%20for%20Brain-Related%20Outcomes&rank=1.

## References

[1] Matthews KA Xu W Gaglioti AH Holt JB Croft JB Mack D et al. Racial and ethnic estimates of Alzheimer’s disease and related dementias in the United States (2015-2060) in adults aged =65 years. *Alzheimers Dement.* (2019) 15:17–24. 10.1016/j.jalz.2018.06.3063 30243772 PMC6333531

[2] Wang XT Wang ZT Hu HY Qu Y Wang M Shen XN et al. Association of subjective cognitive decline with risk of cognitive impairment and dementia: a systematic review and meta-analysis of prospective longitudinal studies. *J Prev Alzheimers Dis.* (2021) 8:277–85. 10.14283/jpad.2021.27 34101784 PMC12280825

[3] Jessen F Amariglio RE van Boxtel M Breteler M Ceccaldi M Chételat G et al. A conceptual framework for research on subjective cognitive decline in preclinical Alzheimer’s disease. *Alzheimers Dement.* (2014) 10:844–52. 24798886 10.1016/j.jalz.2014.01.001 PMC4317324

[4] Márquez F Tarraf W Kuwayama S Valencia DF Stickel AM Morlett-Paredes A et al. Subjective cognitive decline and cognitive change among diverse middle-aged and older Hispanic/Latino adults: results from the Study of Latinos-Investigation of Neurocognitive Aging (SOL-INCA). *Alzheimers Dement.* (2024) 20:7715–28. 10.1002/alz.14232 39234644 PMC11567848

[5] Quiroz YT Solis M Aranda MP Arbaje AI Arroyo-Miranda M Cabrera LY et al. Addressing the disparities in dementia risk, early detection and care in Latino populations: highlights from the second Latinos &. Alzheimer’s Symposium. *Alzheimers Dement.* (2022) 18:1677–86. 10.1002/alz.12589 35199931 PMC9399296

[6] Administration for Community Living *2020 Profile of Hispanic Americans age 65 and Older.* Washington, DC: US Department of Health and Human Services (2020).

[7] American College of Sports Medicine position stand Chodzko-Zajko WJ Proctor DN Fiatarone Singh MA Minson CT Nigg CR et al. Exercise and physical activity for older adults. *Med Sci Sports Exerc.* (2009) 41:1510–30. 10.1249/MSS.0b013e3181a0c95c 19516148

[8] Halloway S Wilbur J Schoeny ME Barnes LL. The relation between physical activity and cognitive change in older Latinos. *Biol Res Nurs.* (2017) 19:538–48. 10.1177/1099800417715115 28658973 PMC5599311

[9] Wilbur J Marquez DX Fogg L Wilson RS Staffileno BA Hoyem RL et al. The relationship between physical activity and cognition in older Latinos. *J Gerontol B Psychol Sci Soc Sci.* (2012) 67:525–34. 10.1093/geronb/gbr137 22321957

[10] Watson KB Carlson SA Gunn JP Galuska DA O’Connor A Greenlund KJ et al. Physical inactivity among adults aged 50 Years and Older - United States, 2014. *MMWR Morb Mortal Wkly Rep.* (2016) 65:954–8. 10.15585/mmwr.mm6536a3 27632143

[11] Kalata C Reyes R Kuhail K Larson JL Chen W. Barriers to physical activity in low-income older adults living in senior housing. *Healthcare.* (2025) 13:1159. 10.3390/healthcare13101159 40427995 PMC12111721

[12] Marquez DX Aguiñaga S Castillo A Hughes SL Der Ananian C Whitt-Glover MC. ¡Ojo! what to expect in recruiting and retaining older latinos in physical activity programs. *Transl Behav Med.* (2020) 10:1566–72. 10.1093/tbm/ibz127 31424554 PMC7796772

[13] Balbim GM Aguiñaga S Ajilore OA Bustamante EE Erickson KI Lamar M et al. The effects of the BAILAMOS™ dance program on physical activity levels and cognition of older Latino adults: a pilot study. *J Aging Health.* (2022) 34:25–40. 10.1177/08982643211020996 34027686

[14] Aguiñaga S Kaushal N Balbim GM Wilson RS Wilbur JE Hughes S et al. Latin dance and working memory: the mediating effects of physical activity among middle-aged and older latinos. *Front Aging Neurosci.* (2022) 14:755154. 35493932 10.3389/fnagi.2022.755154 PMC9051326

[15] Marquez DX Wilson R Aguiñaga S Vásquez P Fogg L Yang Z et al. Regular latin dancing and health education may improve cognition of late middle-aged and older latinos. *J Aging Phys Act.* (2017) 25:482–9. 10.1123/japa.2016-0049 28095105 PMC5515671

[16] Marquez DX Bustamante EE Aguiñaga S Hernandez R. BAILAMOS™: development, pilot testing, and future directions of a Latin dance program for older latinos. *Health Educ Behav.* (2015) 42:604–10. 10.1177/1090198114543006 25108538

[17] Aguiñaga S Marquez DX. Feasibility of a Latin dance program for older latinos with mild cognitive impairment. *Am J Alzheimers Dis Other Demen.* (2017) 32:479–88. 10.1177/1533317517719500 28683560 PMC10852848

[18] Der Ananian C Smith-Ray R Meacham B Shah A Hughes S. Translation of fit & strong! for use by hispanics with arthritis: a feasibility trial of ¡En Forma y Fuerte! *J Aging Phys Act.* (2017) 25:628–38. 10.1123/japa.2016-0256 28290760 PMC8171433

[19] Hughes SL Seymour RB Campbell R Pollak N Huber G Sharma L. Impact of the fit and strong intervention on older adults with osteoarthritis. *Gerontologist.* (2004) 44:217–28. 10.1093/geront/44.2.217 15075418

[20] Haase KR Sirois AC Detwyler D Kardeh B Peacock S Cosco TD et al. Facilitators and barriers faced by community organizations supporting older adults during the COVID-19 pandemic. *BMC Geriatr.* (2025) 25:204. 10.1186/s12877-025-05816-w 40155806 PMC11951528

[21] Lekamwasam R Lekamwasam S. Effects of COVID-19 pandemic on health and wellbeing of older people: a comprehensive review. *Ann Geriatr Med Res.* (2020) 24:166–72. 10.4235/agmr.20.0027 32752587 PMC7533189

[22] Granet J Peyrusqué E Ruiz F Buckinx F Abdelkader LB Dang-Vu TT et al. Web-Based physical activity interventions are feasible and beneficial solutions to prevent physical and mental health declines in community-dwelling older adults during isolation periods. *J Gerontol A Biol Sci Med Sci.* (2023) 78:535–44. 10.1093/gerona/glac127 35675174 PMC9384240

[23] Geraedts H Zijlstra A Bulstra SK Stevens M Zijlstra W. Effects of remote feedback in home-based physical activity interventions for older adults: a systematic review. *Patient Educ Couns.* (2013) 91:14–24. 10.1016/j.pec.2012.10.018 23194823

[24] Nasreddine ZS Phillips NA Bédirian V Charbonneau S Whitehead V Collin I et al. The Montreal Cognitive Assessment, MoCA: a brief screening tool for mild cognitive impairment. *J Am Geriatr Soc.* (2005) 53:695–9. 10.1111/j.1532-5415.2005.53221.x 15817019

[25] Yang C Wang L Hu H Dong X Wang Y Yang F. Montreal cognitive assessment: seeking a single cutoff score may not be optimal. *Evid Based Complement Alternat Med.* (2021) 2021:9984419. 10.1155/2021/9984419 34616484 PMC8487840

[26] Estrella ML Tarraf W Kuwayama S Gallo LC Salazar CR Stickel AM et al. Associations of allostatic load with level of and change in cognitive function among middle-aged and older Hispanic/Latino adults: the study of latinos-investigation of neurocognitive aging (SOL-INCA). *J Alzheimers Dis.* (2024) 99:1047–64. 10.3233/JAD-230796 38758999 PMC11343490

[27] Harris PA Taylor R Minor BL Elliott V Fernandez M O’Neal L et al. The REDCap consortium: building an international community of software platform partners. *J Biomed Inform.* (2019) 95:103208. 10.1016/j.jbi.2019.103208 31078660 PMC7254481

[28] Harris PA Taylor R Thielke R Payne J Gonzalez N Conde JG. Research electronic data capture (REDCap)–a metadata-driven methodology and workflow process for providing translational research informatics support. *J Biomed Inform.* (2009) 42:377–81. 10.1016/j.jbi.2008.08.010 18929686 PMC2700030

[29] Marquez DX Tellez M Ocampo-Mota J Jaldin MA Hughes S Ajilore O et al. C.E.R.E.B.R.O.: a home-based physical activity study for older Latino adults. *Contemp Clin Trials Commun.* (2025) 44:101436. 10.1016/j.conctc.2025.101436 39931141 PMC11808524

[30] Braun V Clarke V. Using thematic analysis in psychology. *Qual Res Psychol.* (2006) 3:77–101. 10.1191/1478088706qp063oa

[31] Knechel NA. The challenges of enrolling older adults into intervention studies. *Yale J Biol Med.* (2013) 86:41–7. 23482244 PMC3584494

[32] Teufel-Shone NI Schwartz AL Hardy LJ de Heer HD Williamson HJ Dunn DJ et al. Supporting new community-based participatory research partnerships. *Int J Environ Res Public Health.* (2019) 16:44. 10.3390/ijerph16010044 30585213 PMC6338906

[33] Grape A Rhee H Wicks M Tumiel-Berhalter L Sloand E. Recruitment and retention strategies for an urban adolescent study: lessons learned from a multi-center study of community-based asthma self-management intervention for adolescents. *J Adolesc.* (2018) 65:123–32. 10.1016/j.adolescence.2018.03.004 29587184 PMC5932256

[34] Ojo EO Thiamwong L. Recruitment strategies for a technology-based fall risk assessment research study among community-dwelling older adults during a global pandemic. *J Gerontol Nurs.* (2024) 50:18–23. 10.3928/00989134-20240809-05 39194324 PMC11382612

[35] Pellegrini CA Wilcox S DeVivo KE Jamieson S. Recruitment and retention strategies for underrepresented populations and adults with arthritis in behavioral interventions: a scoping review. *Arthritis Care Res.* (2023) 75:1996–2010. 10.1002/acr.25098 36752353 PMC12131163

[36] Lim JY Yu H Kwon YE Do JG Hwang JH. Feasibility of digital technology-supported home exercise intervention for health promotion in community-dwelling older adults: a pilot randomized controlled trial. *Heliyon.* (2024) 10:e24933. 10.1016/j.heliyon.2024.e24933 38333828 PMC10850410

[37] Zlatar ZZ Greenwood-Hickman MA Lujan LNM Cooper J Florez-Acevedo S Marquez DX et al. Feasibility and cultural adaptation of a community-engaged physical activity intervention for hispanic older adults: pilot study. *JMIR Form Res.* (2025) 9:e65489. 10.2196/65489 40424571 PMC12154937

[38] Bronas UG Marquez DX Fritschi C Petrarca K Kitsiou S Ajilore O et al. Ecological momentary intervention to replace sedentary time with physical activity to improve executive function in midlife and older latino adults: pilot randomized controlled trial. *J Med Internet Res.* (2024) 26:e55079. 10.2196/55079 39235836 PMC11413544

[39] Wilson J Heinsch M Betts D Booth D Kay-Lambkin F. Barriers and facilitators to the use of e-health by older adults: a scoping review. *BMC Public Health.* (2021) 21:1556. 10.1186/s12889-021-11623-w 34399716 PMC8369710

[40] Eakin E Reeves M Winkler E Lawler S Owen N. Maintenance of physical activity and dietary change following a telephone-delivered intervention. *Health Psychol.* (2010) 29:566–73. 10.1037/a0021359 20954778

[41] Di Lorito C Bosco A Booth V Goldberg S Harwood RH Van der Wardt V. Adherence to exercise interventions in older people with mild cognitive impairment and dementia: a systematic review and meta-analysis. *Prev Med Rep.* (2020) 19:101139. 10.1016/j.pmedr.2020.101139 32793408 PMC7414005

[42] Docu Axelerad A Stroe AZ Muja LF Docu Axelerad S Chita DS Frecus CE et al. Benefits of tango therapy in alleviating the motor and non-motor symptoms of Parkinson’s disease patients-a narrative review. *Brain Sci.* (2022) 12:448. 10.3390/brainsci12040448 35447980 PMC9031475

[43] Killingback C Tsofliou F Clark C. Older people’s adherence to community-based group exercise programmes: a multiple-case study. *BMC Public Health.* (2017) 17:115. 10.1186/s12889-017-4049-6 28122532 PMC5264485

[44] Cintoli S Spadoni G Giuliani V Nicoletti V Del Prete E Frosini D et al. A pilot study investigating the effectiveness, appreciation, and feasibility of a cognitive stimulation program in dementia patients: online versus face-to-face. *Front Psychol.* (2025) 16:1561157. 10.3389/fpsyg.2025.1561157 40260005 PMC12009930

[45] Fields J Cemballi AG Michalec C Uchida D Griffiths K Cardes H et al. In-Home technology training among socially isolated older adults: findings from the tech allies program. *J Appl Gerontol.* (2021) 40:489–99. 10.1177/0733464820910028 32141373

[46] Mehra S van den Helder J Visser B Engelbert RHH Weijs PJM Kröse BJA. Evaluation of a blended physical activity intervention for older adults: mixed methods study. *J Med Internet Res.* (2020) 22:e16380. 10.2196/16380 32459652 PMC7413279

[47] Dagenais M Krajnovic A Galway S Gammage K. Instructors’ perceptions and experiences of teaching online exercise classes to older adults: a qualitative study. *J Aging Phys Act.* (2023) 32:124–37. 10.1123/japa.2022-0434 37883633

