# Supplementary material for: Feasibility and acceptability of two remote physical activity programs for older Latino adults: the C.E.R.E.B.R.O. study, a pilot randomized trial
Source: Front Med (Lausanne). 2026 Aug 28;13:1862017. doi: 10.3389/fmed.2026.1862017 (PMC13561821; doi:10.3389/fmed.2026.1862017)
Supplement: Supplementary file 1 [file Data_Sheet_1.pdf]

Descripción general de la tableta

Diagrama 1.1

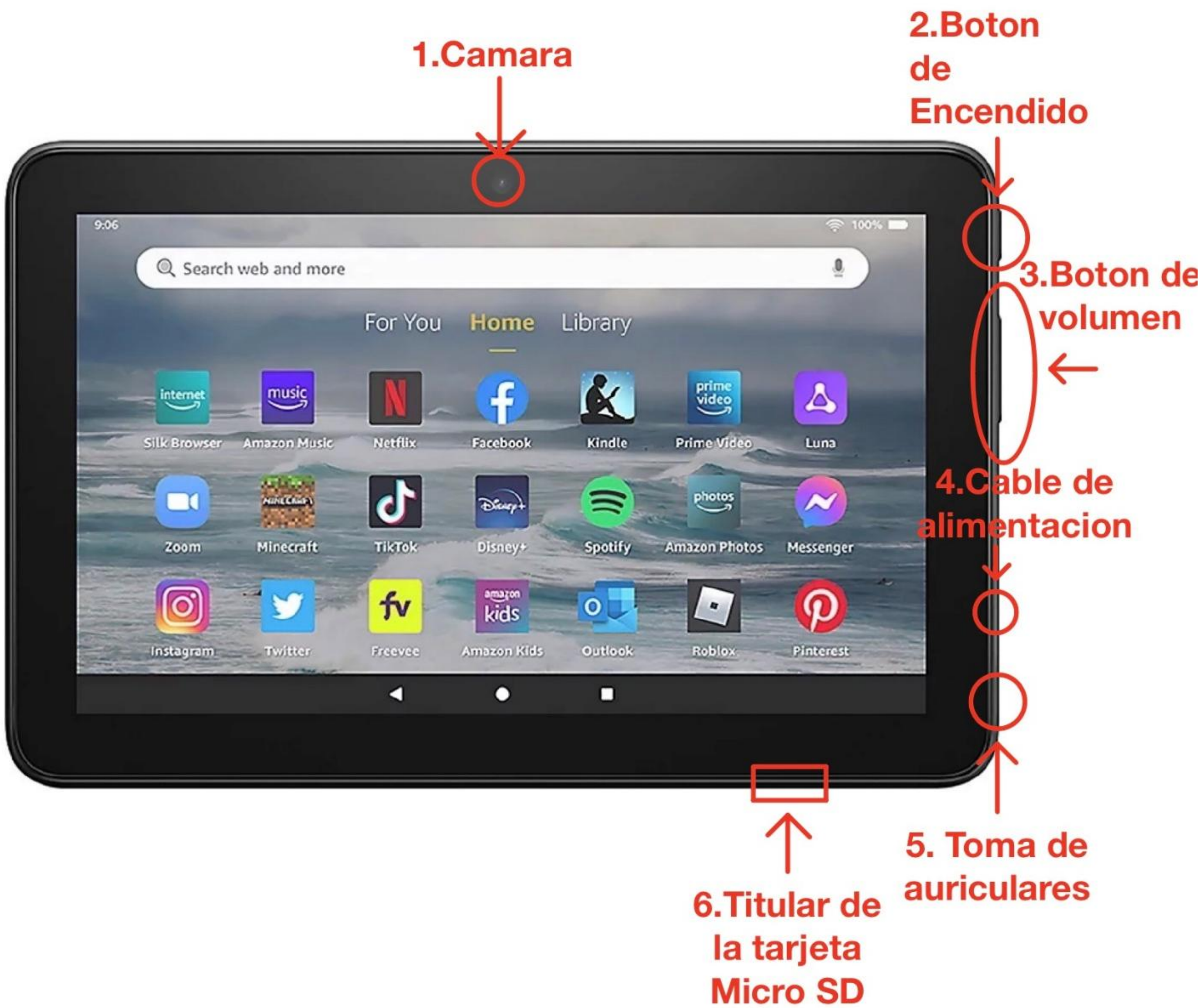

1. Cámara
2. Botón de encendido
3. Botón de volumen
4. Entrada del cable de alimentación
5. Toma de auriculares
6. Titular de la tarjeta Micro SD

Diagrama 1.2

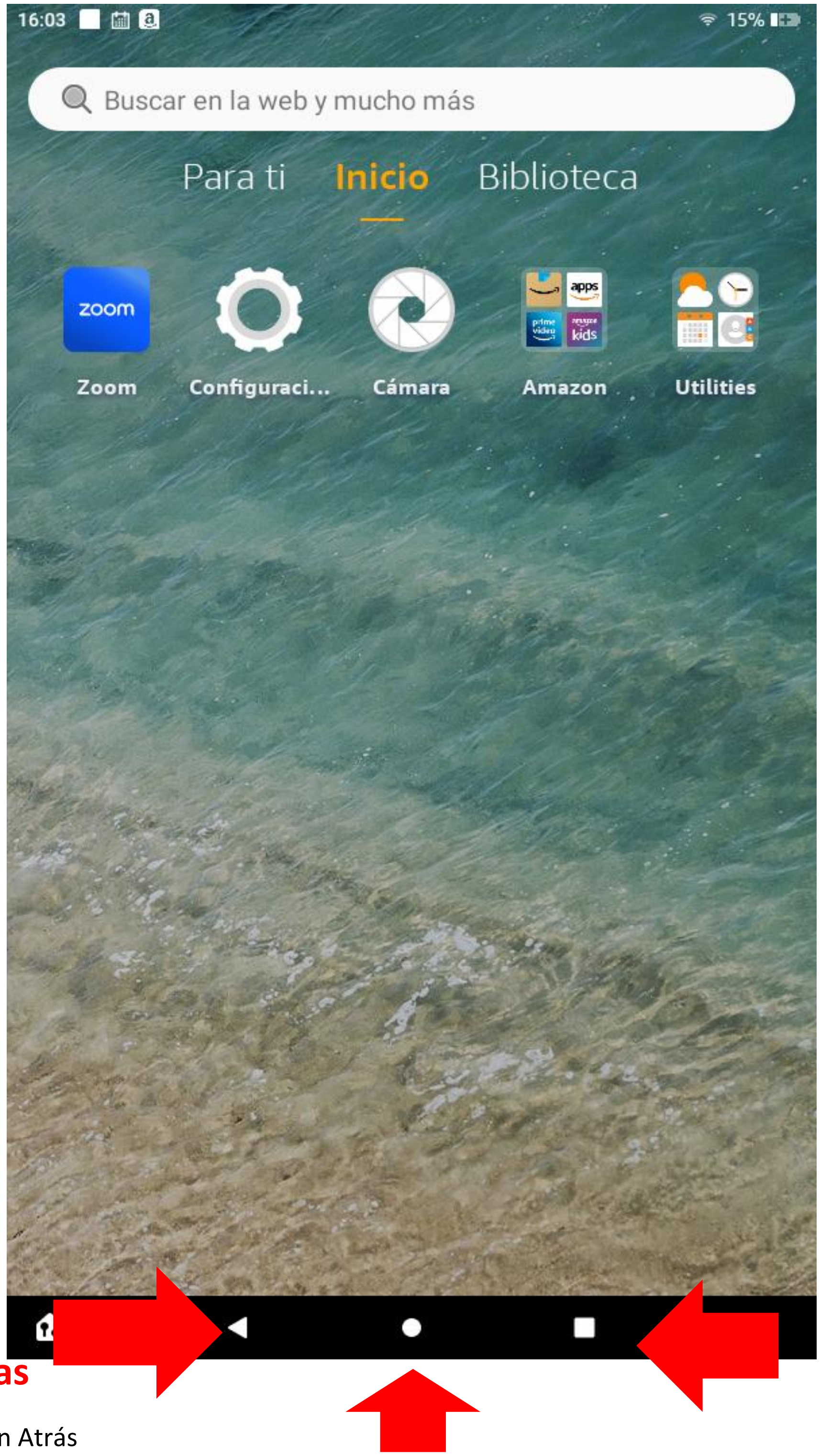

2. Boton Atras

1. Aplicaciones recientes

3. Boton de inicio

1. Botón Atrás
2. Botón de inicio
3. Aplicaciones recientes

Configuración de wifi

1. Una manera fácil de configurar la tableta Amazon 7 en el Wi-Fi/punto de acceso de su hogar es encender la tableta haciendo clic en el botón de encendido número 2 en el diagrama 1.1

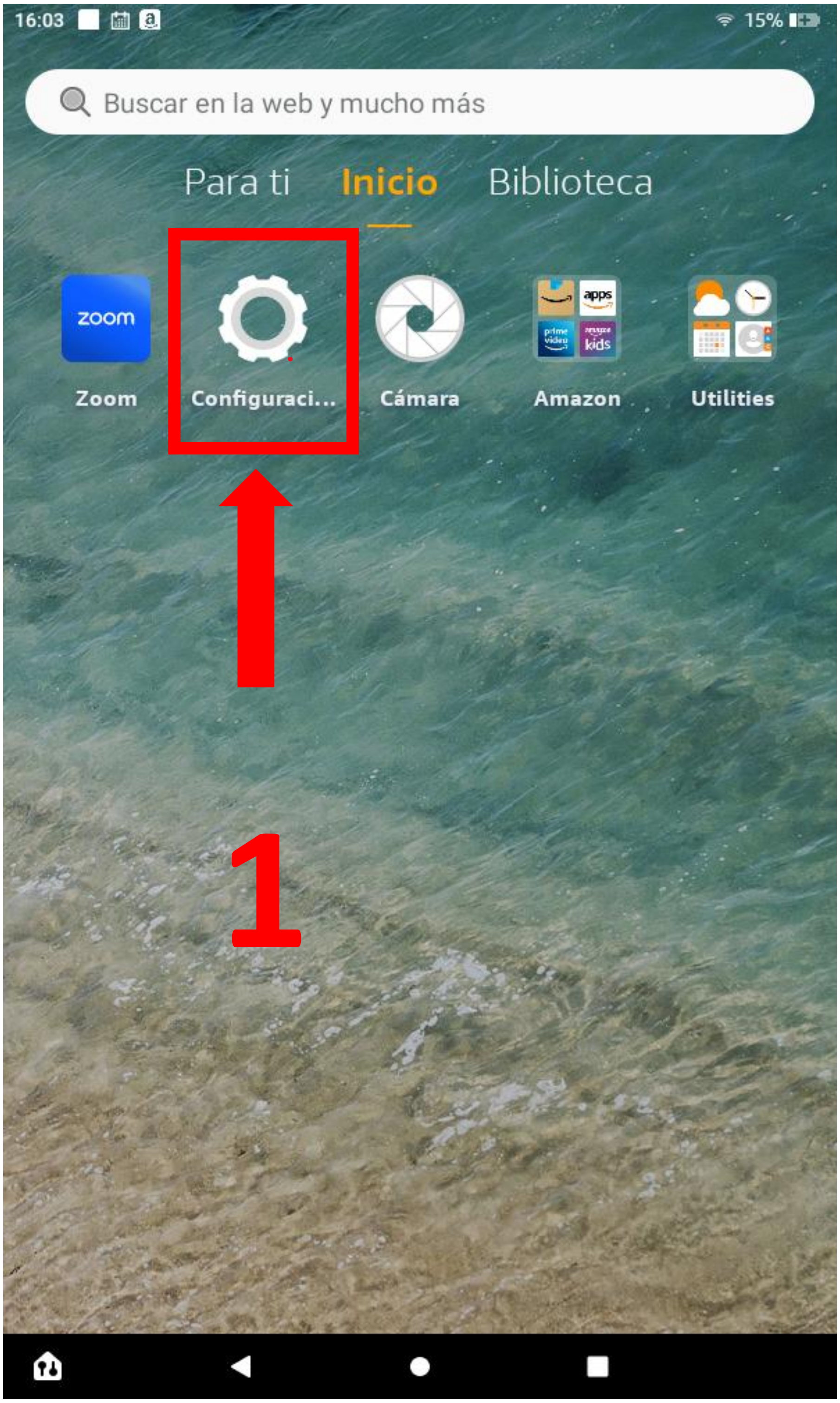

2. Después de abrir la tableta y, en la pantalla de inicio, haga clic en la aplicación de configuración

3. Una vez en los ajustes pulsa en Internet

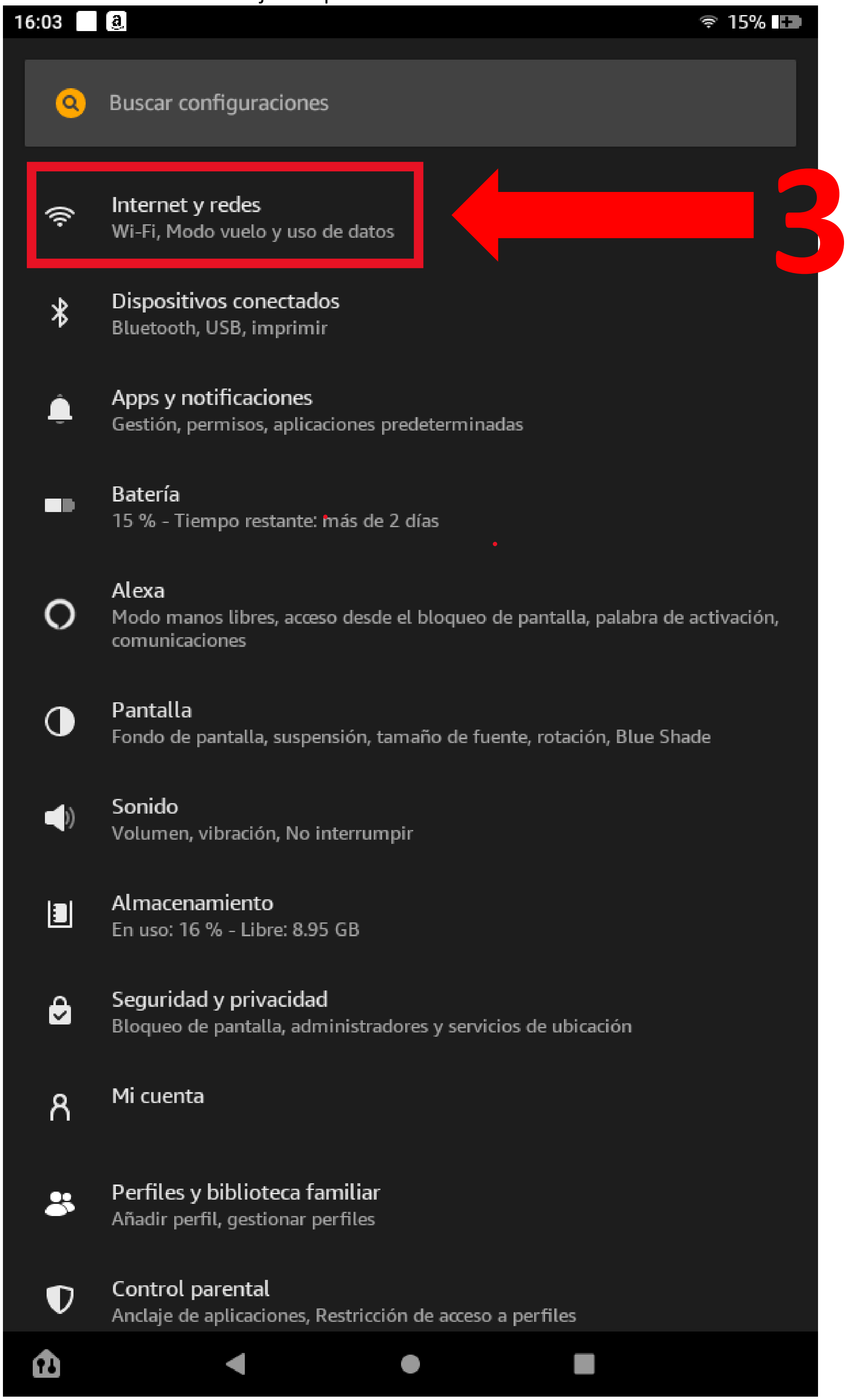

4. Haga clic en Preferencias de Wi-Fi

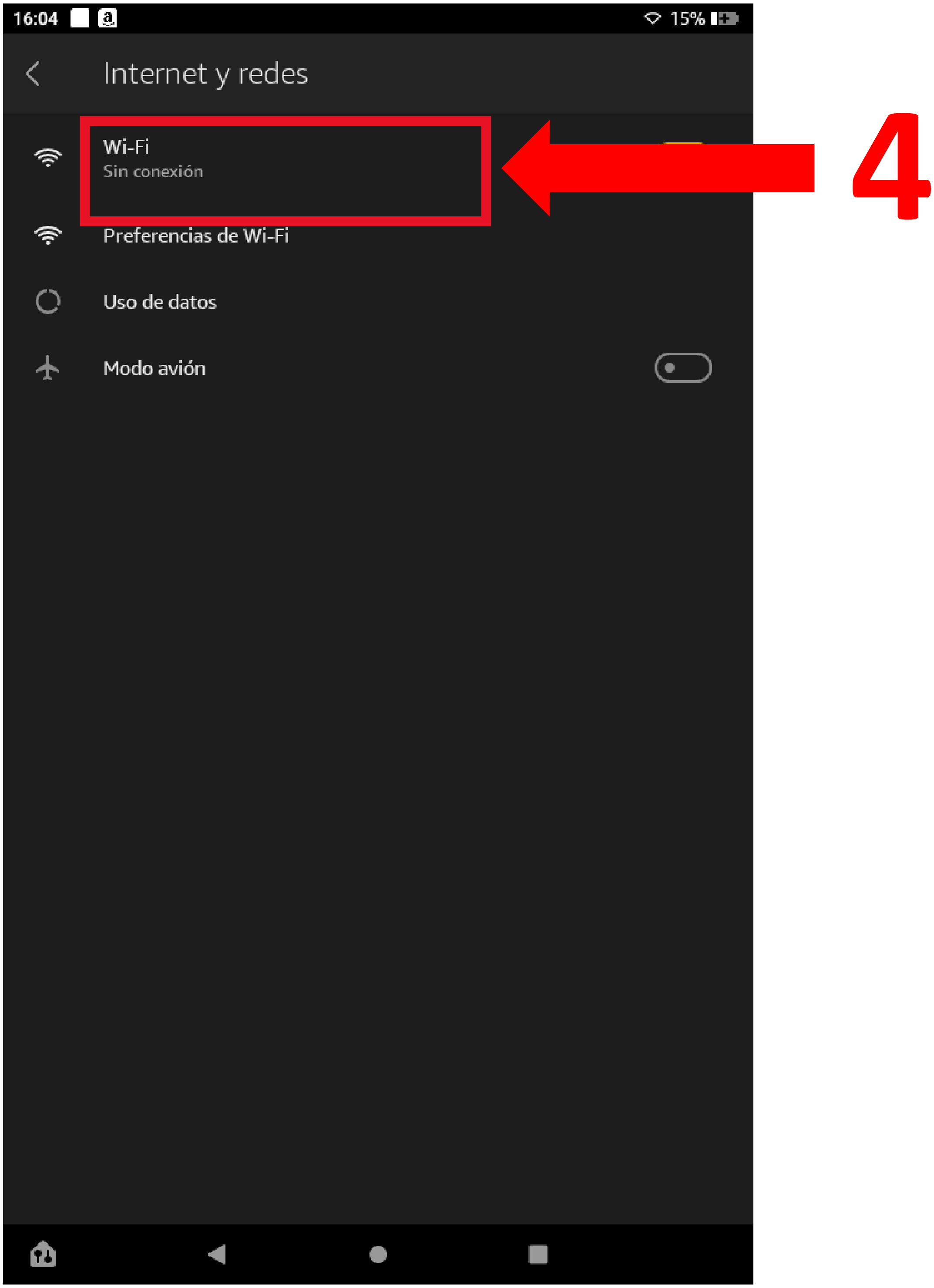

5. Una vez que esté en las preferencias de Wi-Fi, haga clic en el Wi-Fi de su hogar e ingrese su contraseña para conectarse

¿Cómo puedo acceder a la clase por Zoom?

1. Abra la tableta haciendo clic en el botón de encendido #2 en el diagrama 1.1
2. Una vez en la pantalla de inicio, haga clic en la aplicación Zoom

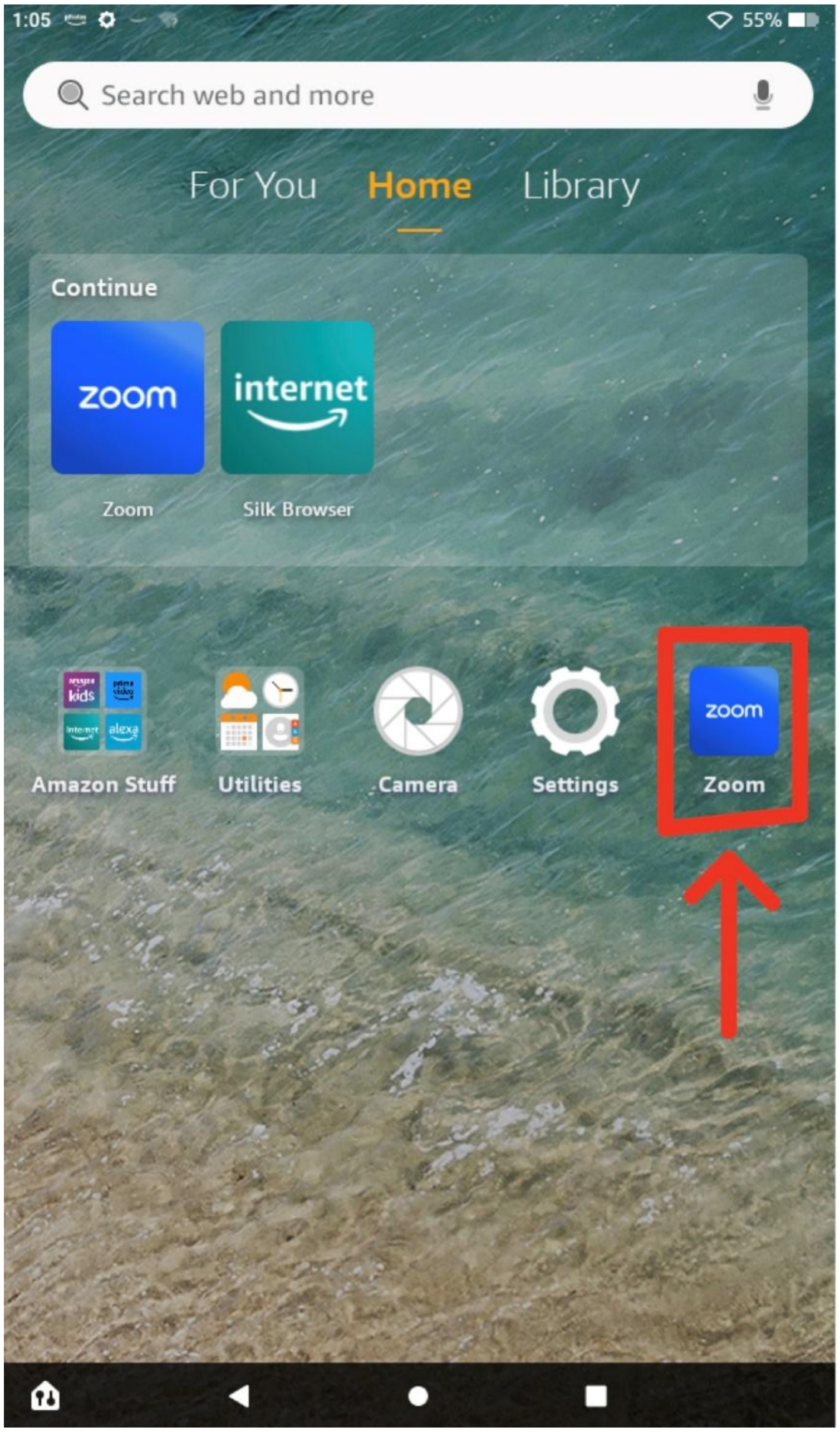

3. Haga clic en "Iniciar sesión". Introduzca su correo electrónico y contraseña de estudio. Encontrará esta clave en la parte posterior de la tableta.

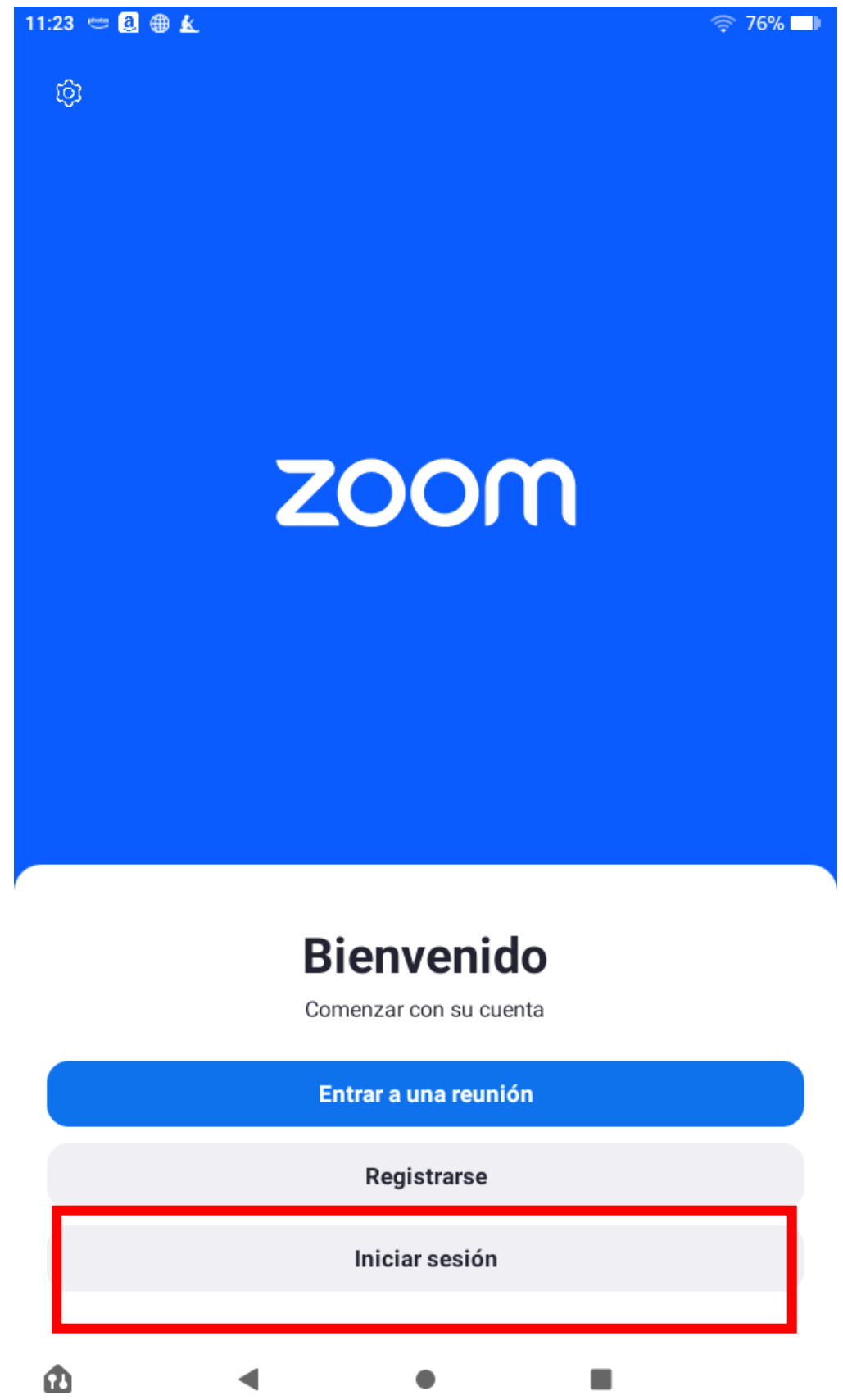

4. Una vez en la aplicación, se le presentará la imagen a continuación, haga clic en unirse a la reunión

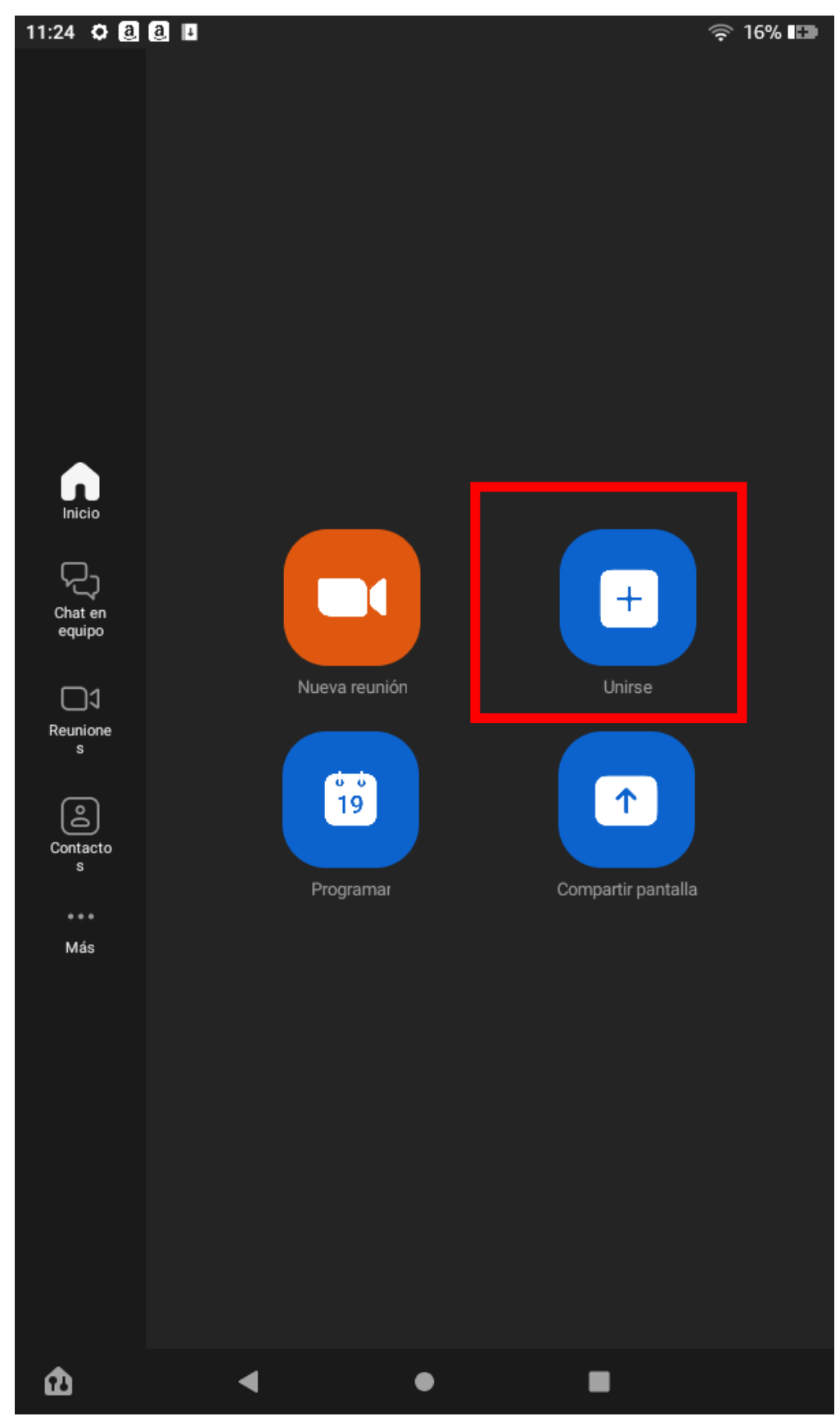

5. Ingrese la ID de la reunión que se encuentra en la parte posterior de su tableta y luego haga clic en Unirse

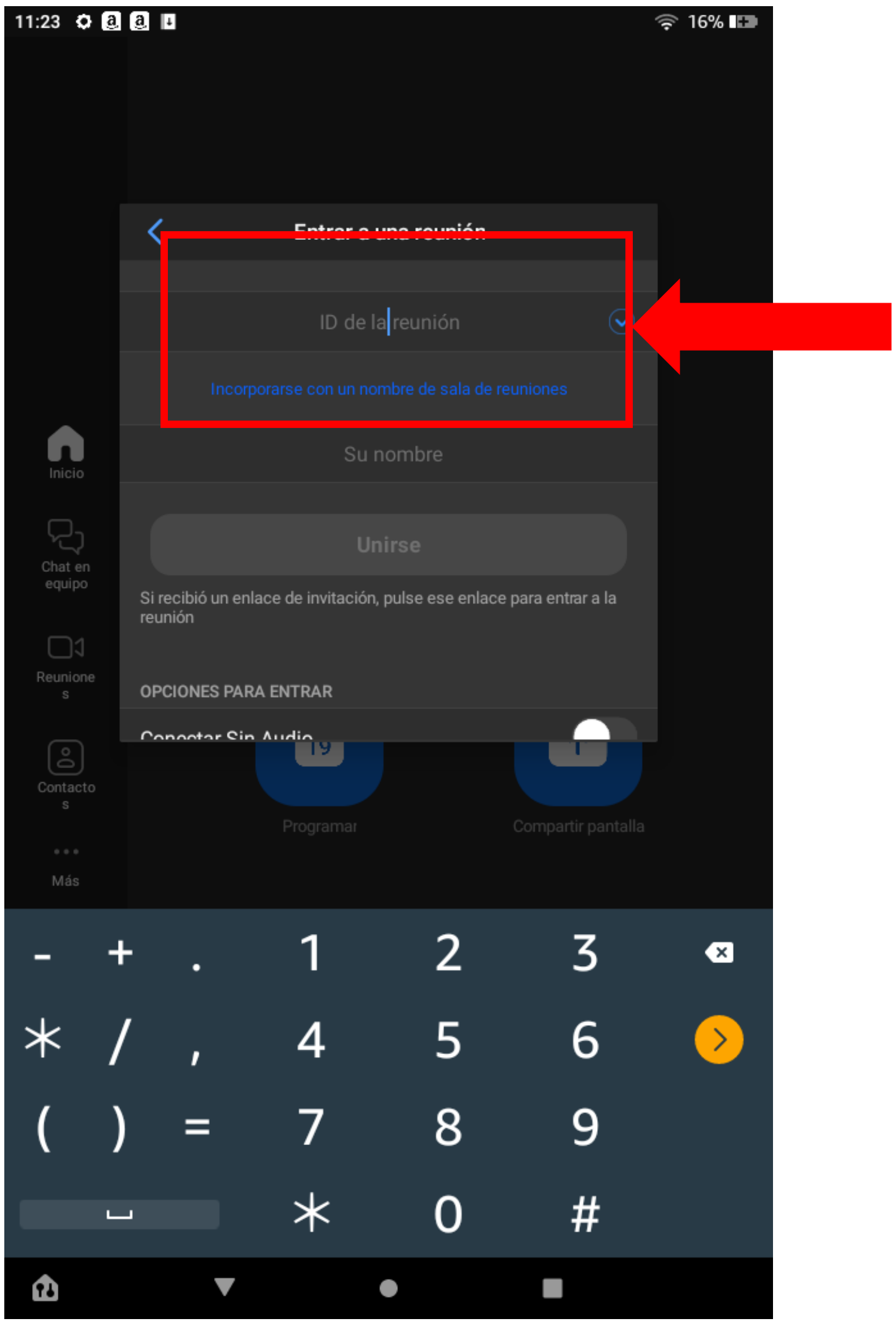

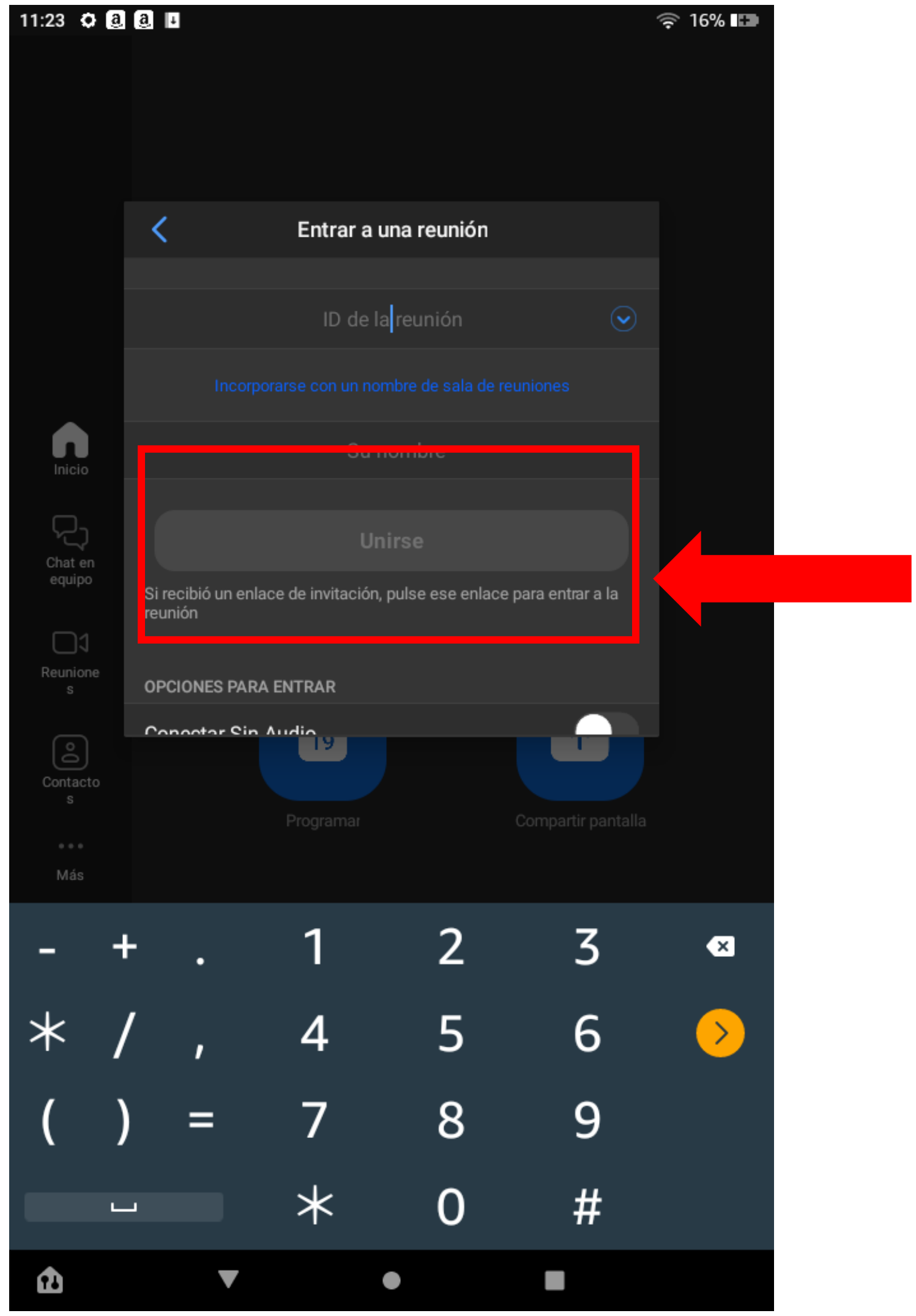

6. Ingrese el código de acceso que se encuentra en la parte posterior de su tableta y luego haga clic en aceptar

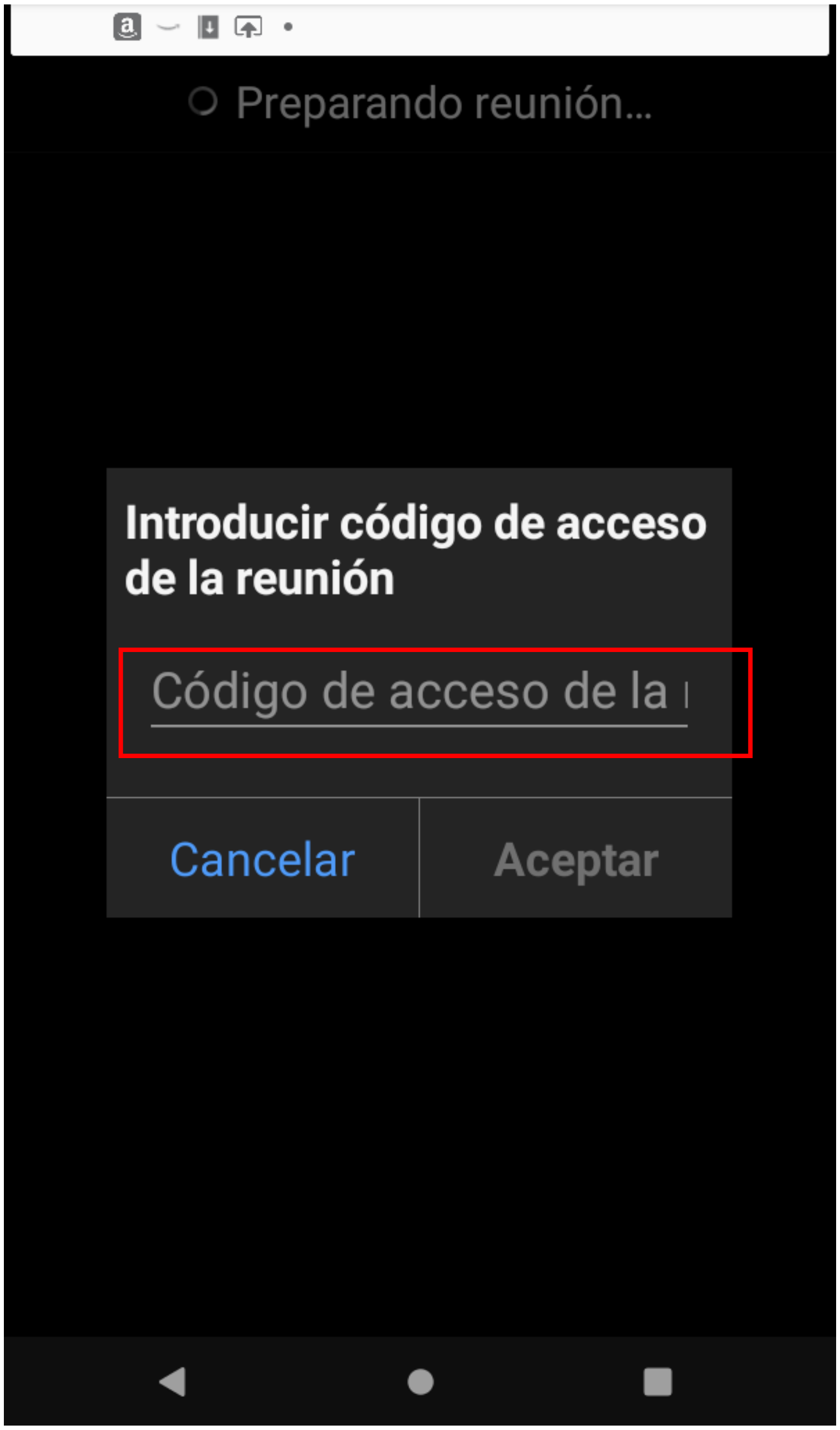

¿Cómo puedo acceder a la clase?

Tu tableta ya tiene instalada la aplicación zoom y la aplicación de correo electrónico Outlook. Hemos creado un correo electrónico de Outlook y una cuneta de Zoom para usted.

1. Haz clic en el icono de Outlook de tu pantalla de inicio.

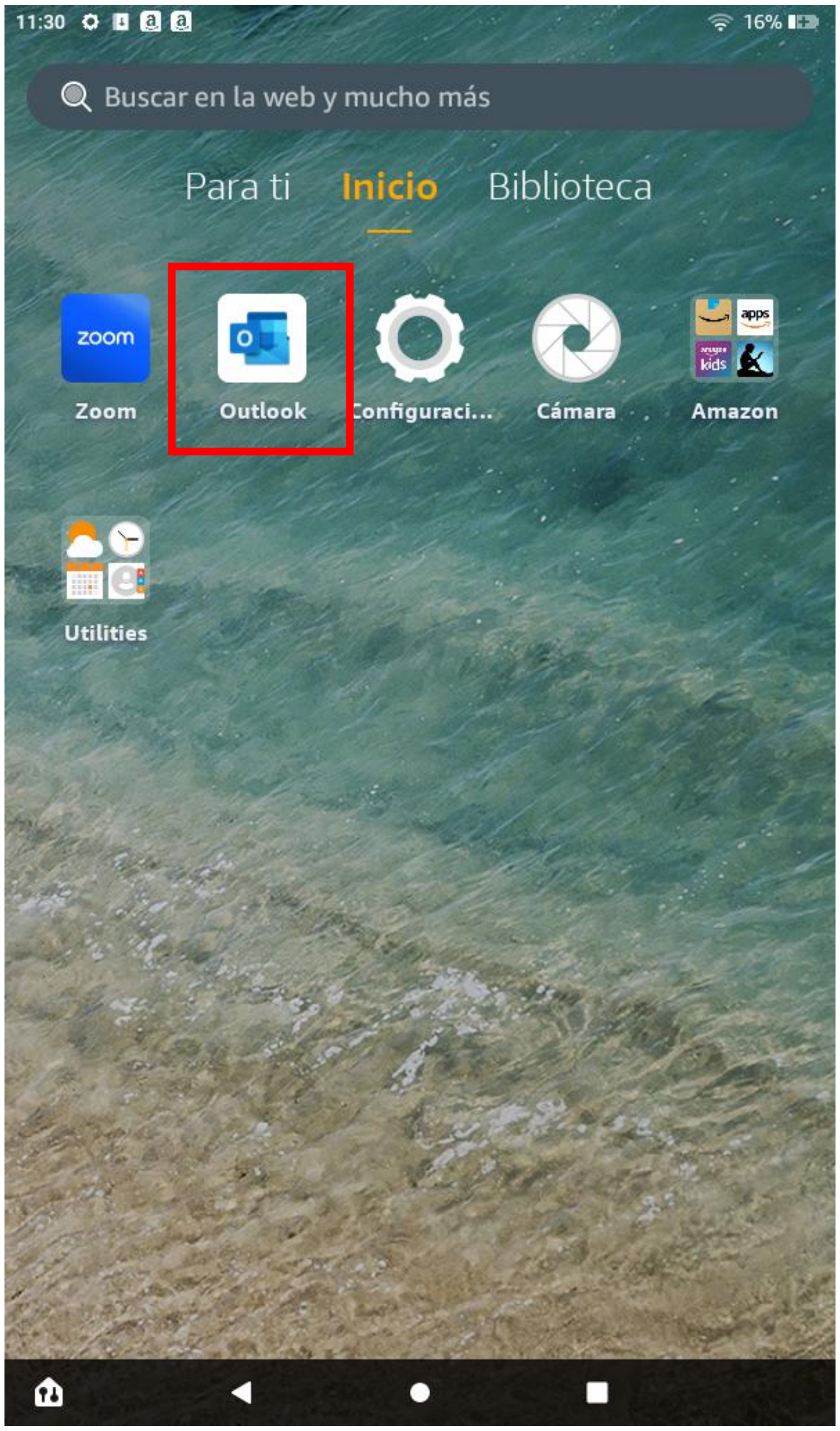

2. En la pantalla de correo electrónico, haz clic en el botón de calendario en la parte inferior de la pantalla.

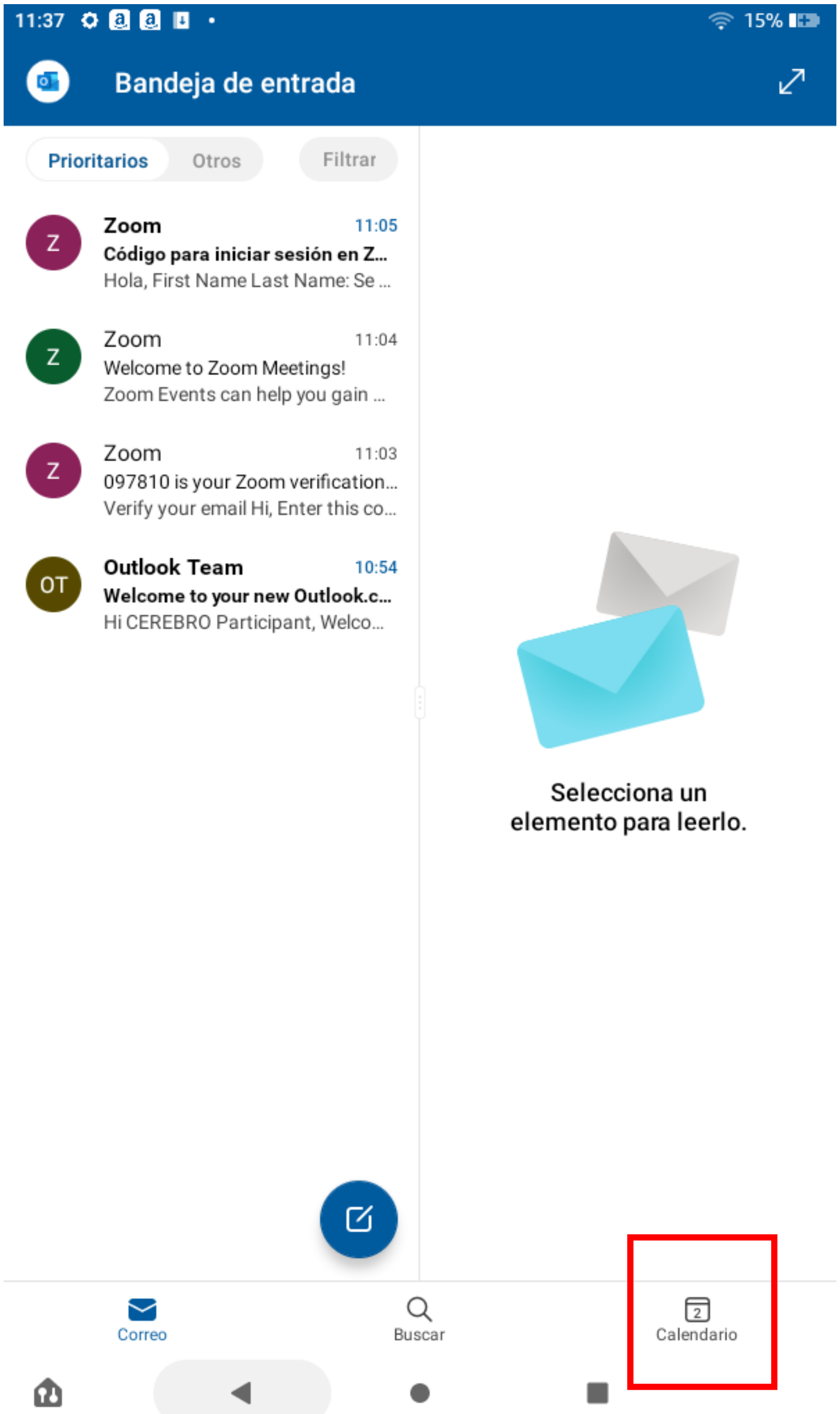

3. Ha abierto su calendario. Verá su clase en el calendario. Para acceder al zoom de la clase haz clic en la clase de hoy.

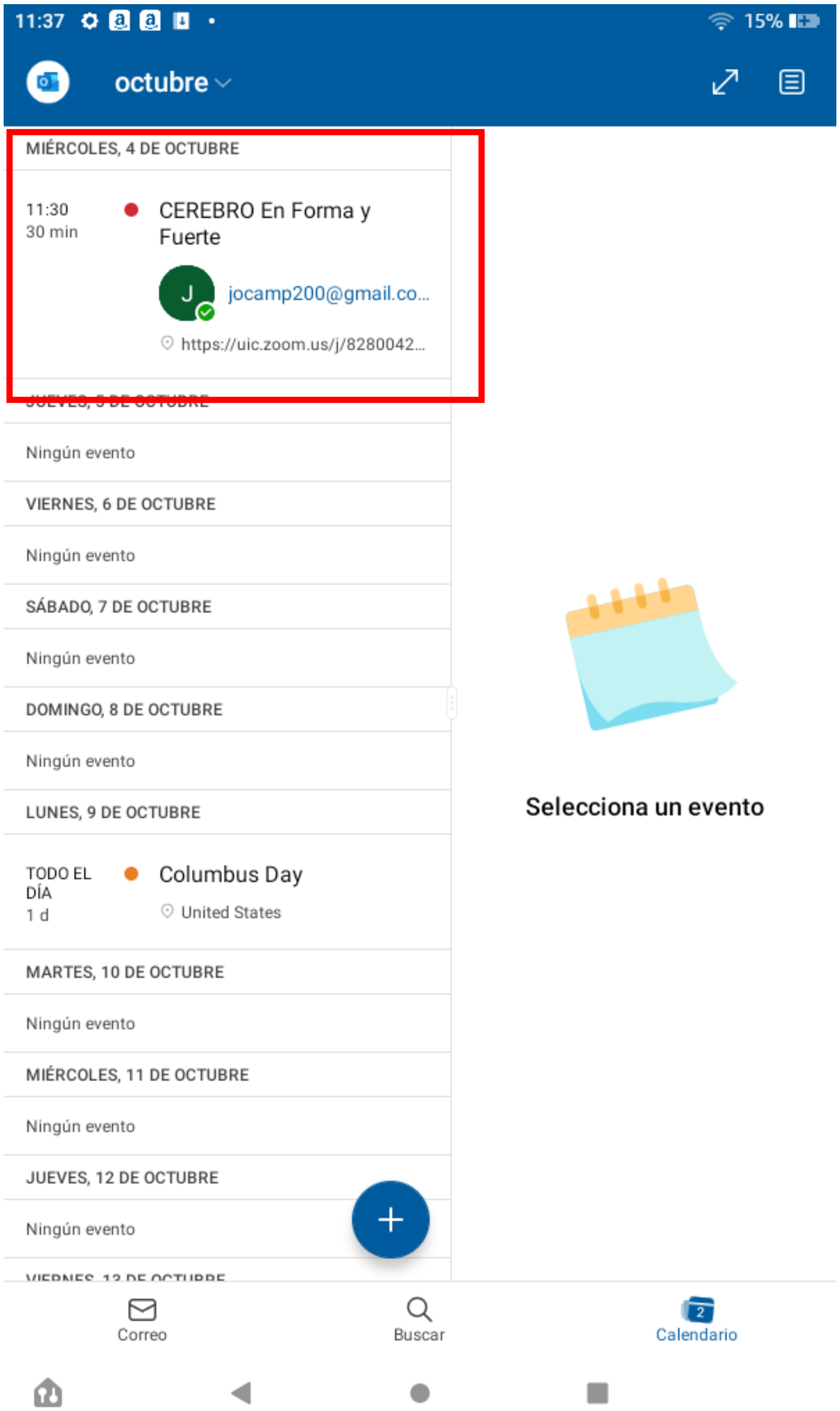

4. Cuando haga clic en la clase, verá un enlace debajo de "ubicación". Haga clic en este enlace. Esto abrirá la sala en la aplicación de zoom de su tableta.

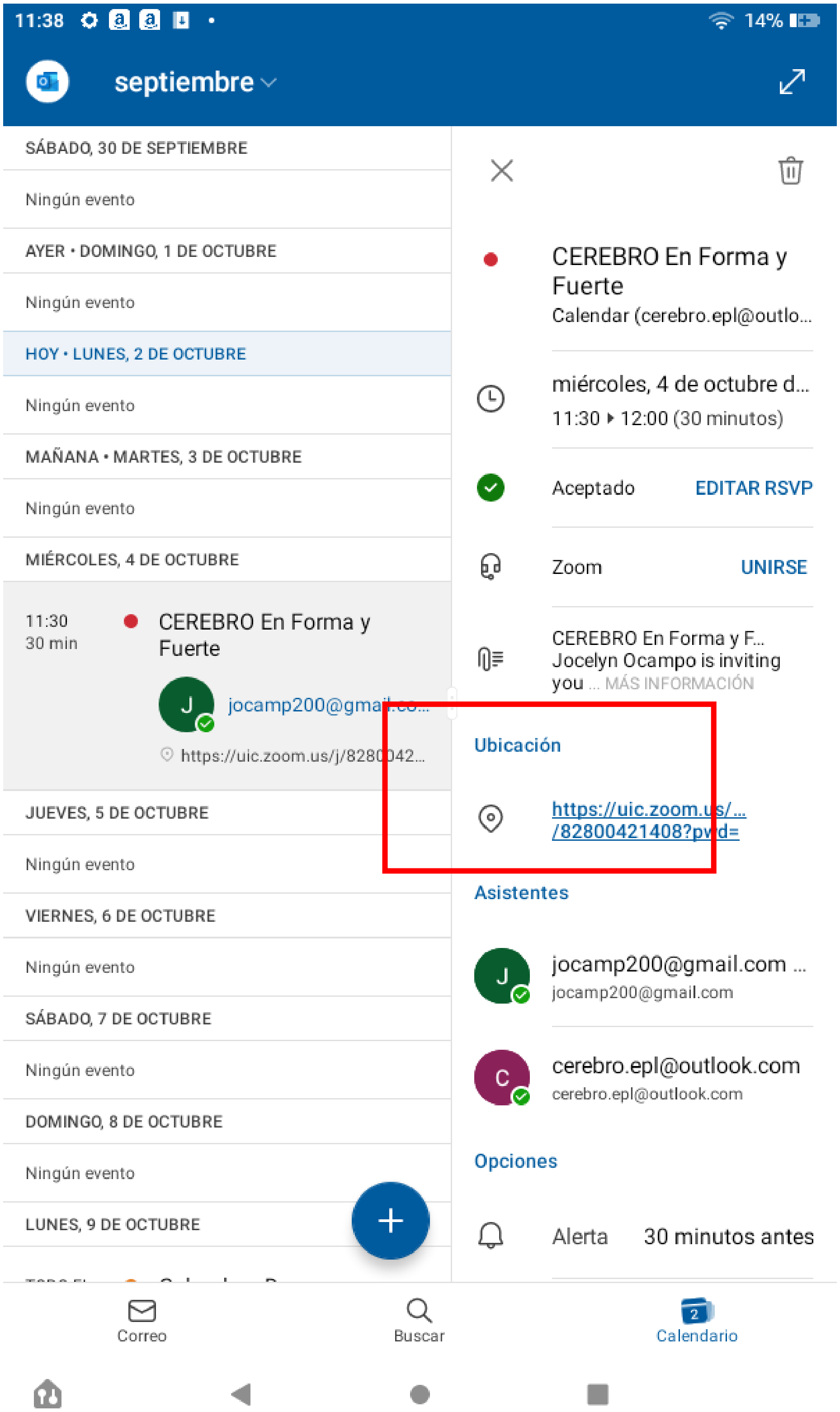
11:38
14%
septiembre
SÁBADO, 30 DE SEPTIEMBRE
Ningún evento
AYER • DOMINGO, 1 DE OCTUBRE
Ningún evento
HOY • LUNES, 2 DE OCTUBRE
Ningún evento
MAÑANA • MARTES, 3 DE OCTUBRE
Ningún evento
MIÉRCOLES, 4 DE OCTUBRE
11:30
30 min
CEREBRO En Forma y Fuerte
jocamp200@gmail.co...
https://uic.zoom.us/j/8280042...
JUEVES, 5 DE OCTUBRE
Ningún evento
VIERNES, 6 DE OCTUBRE
Ningún evento
SÁBADO, 7 DE OCTUBRE
Ningún evento
DOMINGO, 8 DE OCTUBRE
Ningún evento
LUNES, 9 DE OCTUBRE
CEREBRO En Forma y Fuerte
Calendar (cerebro.epl@outlo...
miércoles, 4 de octubre d...
11:30 ▸ 12:00 (30 minutos)
Aceptado
EDITAR RSVP
Zoom
UNIRSE
CEREBRO En Forma y F...
Jocelyn Ocampo is inviting you ... MÁS INFORMACIÓN
Ubicación
https://uic.zoom.us/...
/82800421408?pwd=
Asistentes
jocamp200@gmail.com ...
jocamp200@gmail.com
cerebro.epl@outlook.com
cerebro.epl@outlook.com
Opciones
Alerta
30 minutos antes
Correo
Buscar
Calendario
