# Supplementary material for: Feasibility and acceptability of two remote physical activity programs for older Latino adults: the C.E.R.E.B.R.O. study, a pilot randomized trial
Source: Front Med (Lausanne). 2026 Aug 28;13:1862017. doi: 10.3389/fmed.2026.1862017 (PMC13561821; doi:10.3389/fmed.2026.1862017)
Supplement: Supplementary file 2 [file Supplementary_file_1.pdf]

Baseline participant characteristics (n=38)

**Characteristics**	**BAILAMOS™**	**En Forma y Fuerte!**
**Age, M $\pm$ SD (median)**	70.47$\pm$5.13 (70)	67.32$\pm$5.72 (66)
**Sex, female, n (%)**	17 (89.50)	19 (100)
**Preferred Language, n (%)**		
Spanish	15 (78.90)	16 (84.20)
English	4 (21.10)	3 (15.80)
**Years in school, M $\pm$ SD (median)**	11.05$\pm$4.16 (12)	10.68$\pm$5.20 (12)
**Household income, n (%)**		
$\leq$$10,000	3 (15.80)	6 (31.60)
$10,001-$20,000	5 (26.30)	5 (26.30)
$20,001-$30,000	5 (26.30)	4 (21.10)
$30,001-$40,000	1 (5.30)	1 (5.30)
$40,001-$50,000	3 (15.80)	2 (10.50)
$50,001-$60,000	0	1 (5.30)
$60,001-$70,000	2 (10.50)	
**Sleep, n (%)**		
Less than 7 hours	12 (63.20)	13 (68.40)
7 to 8 hours	5 (26.30)	5 (26.30)
More than 8 hours	1 (10.50)	1 (5.30)
**MoCA Score, M $\pm$ SD**	22.26 $\pm$ 3.33	22.37$\pm$3.72

Notes. *SD*=standard deviation , *M*=mean

MoCA = Montreal Cognitive Assessment (range: 0–30); scores <26 are suggestive of mild cognitive impairment, with lower scores indicating greater cognitive impairment.
